# Anti-Proliferative Potential of Secondary Metabolites from the Marine Sponge *Theonella* sp.: Moving from Correlation toward Causation

**DOI:** 10.3390/metabo11080532

**Published:** 2021-08-10

**Authors:** Kuei-Hung Lai, Bo-Rong Peng, Chun-Han Su, Mohamed El-Shazly, Yi-Long Sun, Ming-Cheng Shih, Yu-Ting Huang, Pei-Tzu Yen, Lung-Shuo Wang, Jui-Hsin Su

**Affiliations:** 1 PhD Program in Clinical Drug Development of Herbal Medicine, College of Pharmacy, Taipei Medical University, Taipei 11031, Taiwan; 2 Graduate Institute of Pharmacognosy, College of Pharmacy, Taipei Medical University, Taipei 11031, Taiwan; 3 Traditional Herbal Medicine Research Center, Taipei Medical University Hospital, Taipei 11031, Taiwan; 4 National Museum of Marine Biology & Aquarium, Pingtung 94450, Taiwan; 610363204@gms.ndhu.edu.tw; 5 Department of Marine Biotechnology and Resources, National Sun Yat-sen University, Kaohsiung 80424, Taiwan; 6 Agricultural Biotechnology Research Center, Academia Sinica, Taipei 11529, Taiwan; d02623001@ntu.edu.tw; 7 Department of Pharmaceutical Biology, German University in Cairo, Cairo 11432, Egypt; mohamed.elshazly@pharma.asu.edu.eg; 8 Department of Pharmacognosy and Natural Products Chemistry, Faculty of Pharmacy, Ain-Shams University, Organization of African Unity Street, Abassia, Cairo 11566, Egypt; 9 Department of Biological Science & Technology, I-Shou University, Kaohsiung 82445, Taiwan; isu10141004m@isu.edu.tw (Y.-L.S.); mchshih@isu.edu.tw (M.-C.S.); 10 Department of Nutrition, I-Shou University, Kaohsiung 82445, Taiwan; ythuang@isu.edu.tw; 11 Department of Chinese Medicine, Sin-Lau Hospital, Tainan 70142, Taiwan; d4848@sinlau.org.tw; 12 The School of Chinese Medicine for Post Baccalaureate, I-Shou University, Kaohsiung 82445, Taiwan

**Keywords:** *Theonella* sp., anti-proliferation, 4-methylene sterols, nucleosides, macrolides, ChemGPS-NP

## Abstract

Marine sponges have been recognized as a rich source of potential anti-proliferative metabolites. Currently, there are two sponge-derived anti-cancer agents (a macrolide and a nucleoside) isolated from the Porifera phylum, suggesting the great potential of this sponge as a rich source for anti-neoplastic agents. To search for more bioactive metabolites from this phylum, we examined the EtOAc extract of *Theonella* sp. sponge. We isolated seven compounds (**1**–**7**), including four 4-methylene sterols (**1**–**4**), two nucleosides (**5** and **6**), and one macrolide (**7**). Among them, theonellasterol L (**1**) was identified for the first time, while 5′-*O*-acetyl-2′-deoxyuridine (**5**) and 5′-*O*-acetylthymidine (**6**) were the first identified deoxyuridine and thymidine derivatives from the sponge *Theonella* sp. These structures were elucidated based on their spectroscopic data. The anti-proliferation activity of compounds **1**–**7** against the MCF-7, MDA-MB-231, T-47D, HCT-116, DLD-1, K562, and Molt 4 cancer cell lines was determined. The results indicated that the 14-/15-oxygenated moiety played an important role in the antiproliferative activity and the macrolide derivatives dominated the anti-proliferative effect of the sponge *Theonella* sp. The in silico analysis, using a chemical global positioning system for natural products (ChemGPS-NP), indicated an anti-proliferative mode of actions (MOA) suggesting the potential applications of the isolated active metabolites as anti-proliferative agents.

## 1. Introduction

Marine sponges are rich sources for natural products and many compounds derived from these organisms have grabbed the attention of the scientific community since they possess complex structures that are difficult to elucidate and synthesize [1,2]. In 1969, cytarabine (Cytosar-U) became the first marine anti-cancer drug to be approved [3]. Thereafter, more than 1600 scientific reports addressed the great potential of sponges as a source of novel cytotoxic metabolites. Currently, there are two sponge-derived anti-cancer compounds in the pipeline. Initial results suggested their potential activity as potent new chemotherapeutic agents with high selectivity index and lower side effects.

The marine sponge *Theonella* sp. belongs to the order Lithistida, which is characterized by a hard skeleton comprised of interlocking spicules of silica [4]. The sponge is globally distributed and contains a variety of metabolites with cytotoxic activity including 4-methylene steroids [5], nucleosides [6], macrolides [7,8,9], cyclopeptides [10,11,12], cyclodepsipeptides [13,14], linear peptides [15], and alkaloids [16,17]. The 4-methylene steroids, characterized by an exocyclic double bond at C-4, were exclusively identified from the genus *Theonella*, suggesting their potential use as chemotaxonomic markers [18]. The cytotoxic properties of these biosynthetically related steroids with the unusual skeleton were studied against breast carcinoma (MCF-7, MDA-MB-231, and T-47D), colon carcinoma (HCT-116 and DLD-1), lung adenocarcinoma (PC9), lymphoma (U937), and leukemia (K562 and Molt 4) [5,18]. Another interesting class of cytotoxic compounds is the sponge-derived nucleosides that exhibited interesting biological activity [19]. The only reported nucleoside isolated from the genus *Theonella* was Kumusine. This nucleoside showed potent cytotoxic effects against leukemia (P388), lung carcinoma (A549), and colon carcinoma (HT-29) [6]. The macrolide analogs identified from the genus *Theonella*, such as swinholides, were screened against several colon carcinomas (HCT-116) [20] hepatocellular carcinoma (HepG2) [8], and papilloma (KB) cells [9]. They exhibited potent cytotoxic activity with an IC_50_ value in the nanomolar range. Further studies revealed that their cytotoxic mechanism of action involved the disruption of the actin/microtubule cytoskeleton [7,20,21]. Interestingly, recent biosynthetic studies pointed out that this class of compounds was most likely to be produced by cyanobacteria living in symbiosis with *Theonella* sp. [7].

The promising cytotoxicity of the identified metabolites from *Theonella* sp. sponge encouraged us to conduct more research aiming to identify interesting bioactive compounds. In the current study, apart from the usual macrolide analog, we isolated four unique 4-methylene steroids, as well as two rarely found nucleoside derivatives. A limited panel of cancer cells was used to assess the anti-proliferative potential of all isolates. The structure-activity relationship (SAR) of the identified 4-methylene steroids was discussed and compared with the previously reported analogs. To predict the possible anti-proliferative mechanism of action, a principal component analysis (PCA)-based computational model was applied to interpret the physical–chemical properties of the isolated compounds in comparison with a series of promising clinical chemotherapeutic agents.

## 2. Results and Discussion

**Figure 1:**
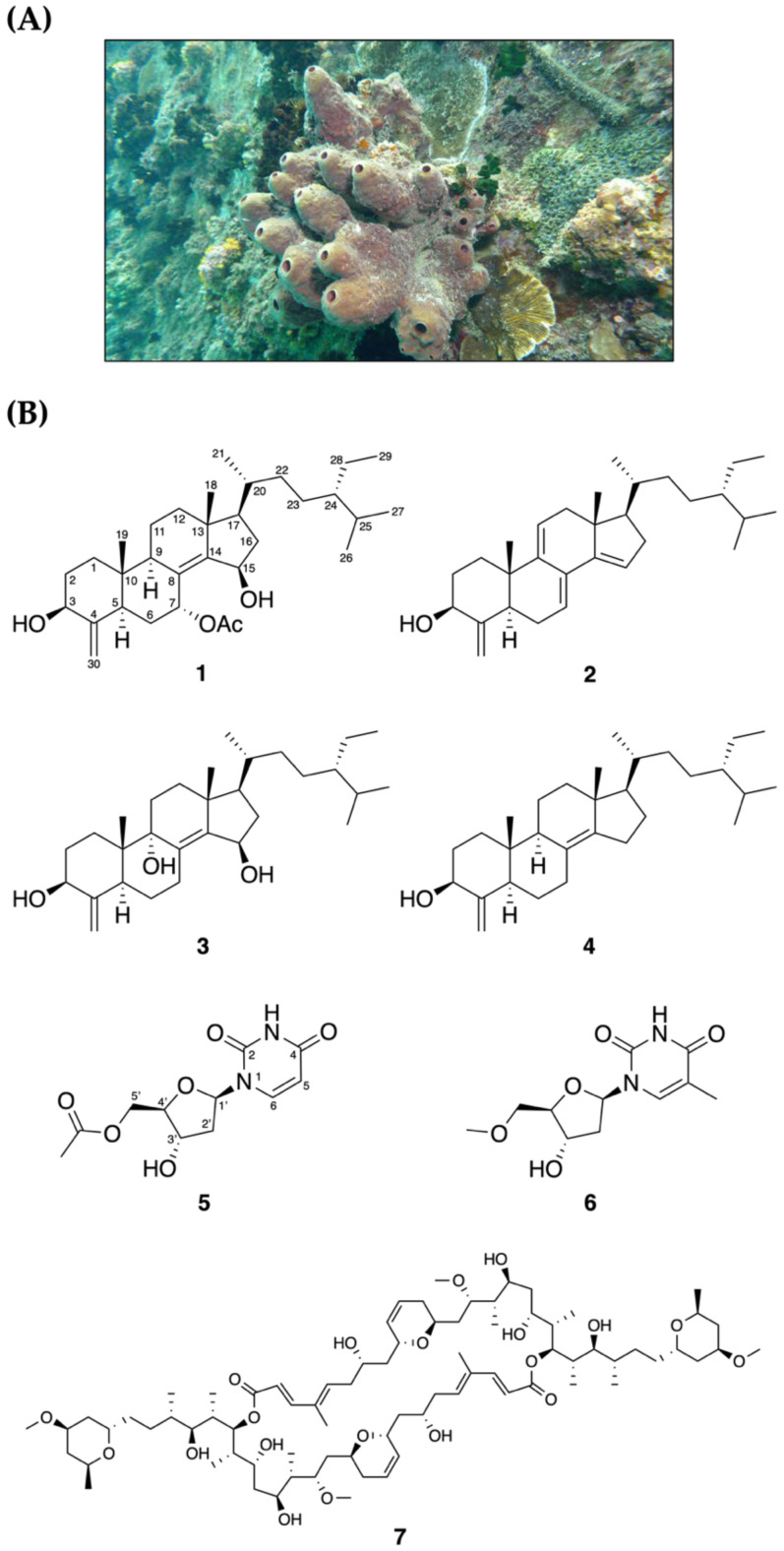
(**A**) *Theonella* sp. sponge in its natural habitat and (**B**) the isolated metabolites.

The freeze-dried material of the marine sponge *Theonella* sp. (Figure 1) was ground and extracted with ethyl acetate (EtOAc) to provide the crude extract. The crude extract was fractionated and purified on a series of normal-phase column chromatography affording seven compounds including theonellasterol L (**1**), theonellasterol B (**2**) [22], theonellasterol E (**3**) [22], theonellasterol (**4**) [23], 5′-*O*-acetyl-2′-deoxyuridine (**5**) [24], 5′-*O*-acetylthymidine (**6**) [25], and swinholide A (**7**) [26]. Theonellasterol L (**1**) was found to be a novel compound belonging to the *Theonella* sp. biomarker group of compounds, 4-methylene sterols. The identified deoxyuridine and thymidine derivatives, 5′-*O*-acetyl-2′-deoxyuridine (**5**) and 5′-*O*-acetylthymidine (**6**), were reported for the first time from this sponge.

Theonellasterol L (**1**) was isolated as a pale-yellow oil. The HRESIMS spectrum of **1** showed a pseudo molecular ion peak at *m*/*z* 523.3768 [M + Na]^+^. The HRESIMS and ^13^C NMR data suggested a molecular formula of C_32_H_52_O_4_, implying seven degrees of unsaturation. The IR spectrum of **1** indicated the presence of hydroxy and ester functionalities based on the absorptions at 3410 and 1712 cm^−1^, respectively.

The gross structure was inferred from the ^13^C NMR and DEPT spectroscopic data (Table 1), which showed signals of thirty carbons including seven methyls, nine sp^3^ methylenes, one sp^2^ methylene, nine sp^3^ methines (including three oxymethines), two sp^3^ quaternary carbons, and three sp^2^ quaternary carbons. The ^1^H NMR spectrum of **1** also revealed the characteristic down-fielded resonances of two olefinic methylene protons (*δ*_H_ 5.93 and 4.80, both s) and three oxygenated methines (*δ*_H_ 5.93, brs; 4.80, d, *J* = 6.0 Hz; and 4.06, m).

**Figure 2:**
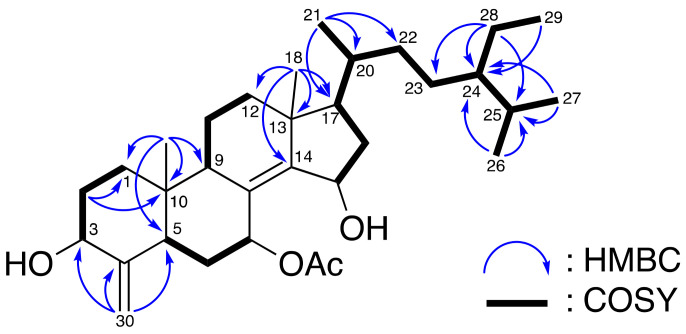
Selected ^1^H–^1^H COSY and HMBC correlations of **1**.

The structure was further illustrated based on the 2D NMR spectra including ^1^H–^1^H COSY and HMBC spectra (Figure 2). The ^1^H–^1^H COSY spectra suggested four partial structures (H-1 to H-3; H-5 to H-7; H-9 to H-12; and H-15 to H-17–H-20 to H-29) of consecutive proton spin systems (Figure 2). The HMBC cross-peaks (from H-2 to C-1 and C-10; from H-30 to C-3, C-4, and C-5; from H-19 to C-1, C-9, and C-10; from H-18 to C-12, C-13, C-14, and C-17; from H-21 to C-17, C-20, and C-22; from H-28 to C-23, C-24, and C-25; from H-29 to C-24; from both H-26 and H-27 to C-24 and C-25) connected the partial structures resulting in the successful establishment of the gross structure of **1** (Figure 2). Compound **1** was found to possess a tetracyclic steroidal skeleton with olefinic methylene at C-4, a double bond at C-8/C-14, two hydroxy groups at C-3 and C-15, and one acetyl group at C-7.

**Figure 3:**
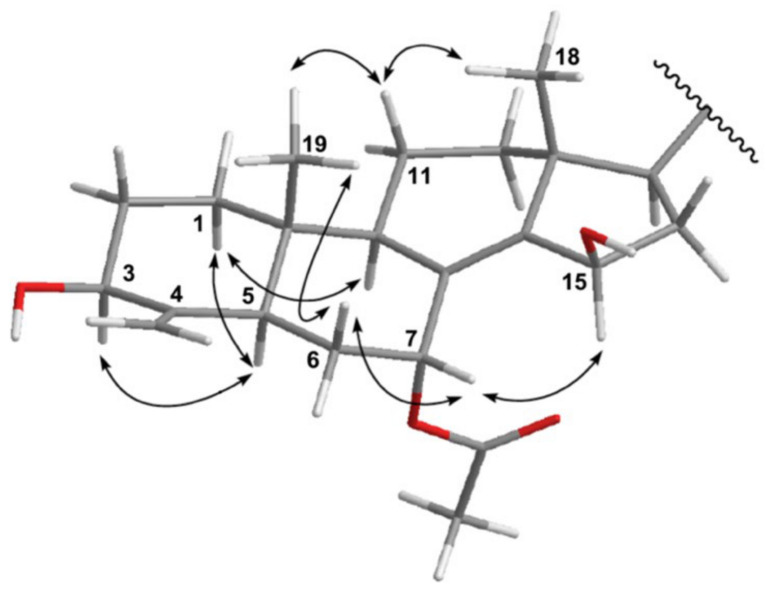
Selected NOESY correlations for **1**.

As shown in Figure 3, the most stable conformation was suggested using molecular mechanics calculations (MM2). The relative configurations of the nine chiral centers at C-3, C-5, C-7, C-9, C-10, C-13, C-15, C-17, and C-20 in **1** were elucidated based on the observed NOESY correlations (Figure 3). Since all naturally occurring 4-methylene sterols showed that H-5 to be *trans* to Me-19, we suggested the *β*-orientation of H_3_-19. Thus, the NOESY correlations between one of the methylene protons at C-11 (*δ*_H_ 1.53) and both methyls (H_3_-18 and H_3_-19), as well as between H_3_-18 and H-20, indicated that these protons were *β*-oriented. H-5 showed a correlation with H-3, revealing a 3-*β* oriented hydroxy group. On the other hand, the α-oriented 17-OAc was suggested based on the NOESY cross-peaks between H_3_-19/H-6*β* and H-6*β*/H-7. Since H-15 did not interact with the H_3_-18, we suggested the *β*-configuration of 15-OH. This 15-*β*-OH configuration could be confirmed by the fact that the α-face of H-15 is closer to H-7 in space (a NOESY correlation between H-15 and H-7 was observed. For the assignment of the side chain at C-24, the H_3_-29 resonance at *δ*_H_ 0.86 suggested a 24*S* configuration rather than 24*R* [27]. Accordingly, the structure of **1** was established and named as Theonellasterol L.

To evaluate the anti-proliferative activity of the isolated secondary metabolites from the marine sponge *Theonella* sp., all obtained compounds were subjected to the MTT antiproliferative assay (Table 2). The used cancer cell lines included MCF-7 and MDA-MB-231 (human breast adenocarcinoma), T-47D (human hormone-dependent breast cancer), HCT-116, and DLD-1 (human colon adenocarcinoma), K562 (human chronic myelogenous leukemia), and Molt 4 (human T lymphoblast, acute lymphoblastic leukemia).

**Figure 4:**
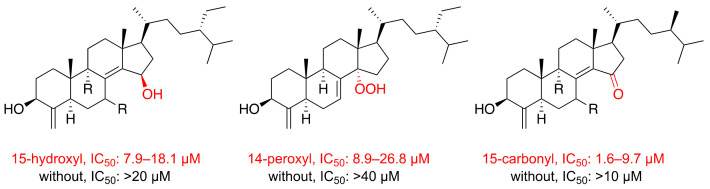
Summary of the structure-activity relationships (SAR) of the anti-proliferative activity of 4-methylene steroids.

By analyzing the results of the 4-methylene sterols (**1**–**4**), it was found that only the derivatives with 14- or 15-oxygenated functionalities showed cytotoxic activity with an IC_50_ value less than 20 μM. The cytotoxic activity based on the specific functionality on the structure (Figure 4) was also reported with the previous reported cytotoxic 4-methylene steroids including theonellasterol K [5] with 14-hydroperoxy oxygenated functionality (IC_50_ 8.94–26.82 μM); 7,15-oxoconicasterol [18] with 15-carbonyl (IC_50_ 4.2–8.8 μM); 15-oxoconicasterol [18] with 15-carbonyl (IC_50_ 2.0–9.7 μM); and 9α-15-oxoconicasterol [18] with 15-carbonyl (IC_50_ 1.6–5.2 μM). On the other hand, the derivatives lacking special functionality showed no cytotoxic activity (IC_50_ > 20 μM) including 7α-hydroxytheonellasterol [28], theonellasterol [5], acetyltheonellasterol [5], acetyldehydroconicasterol [5], conicasterol L [18], and conicasterol [18].

The anti-proliferative results of the isolated nucleosides (**5** and **6**) showed a more potent cytotoxic effect with the IC_50_ ranging from 2.50–18.08 μM. Both nucleosides showed selective activity. Compound **5** showed potent effect (IC_50_ 2.50 μM) against DLD-1 (Human Dukes’s type C colorectal adenocarcinoma cell) but not against HCT-116 (human colon cancer cell line with a mutation in codon 13 of the KRAS proto-oncogene); while **6** affected MDA-MB-231 (breast adenocarcinoma) but not MCF-7 (invasive ductal breast carcinoma) and T-47D (invasive ductal breast carcinoma). The only isolated macrolide, swinholide A (**7**), showed the most potent inhibitory effect against all cancer lines at the nanomolar level (except for MDA-MB-231). The cytotoxic results of **7** were in agreement with the previously published results [20].

**Figure 5:**
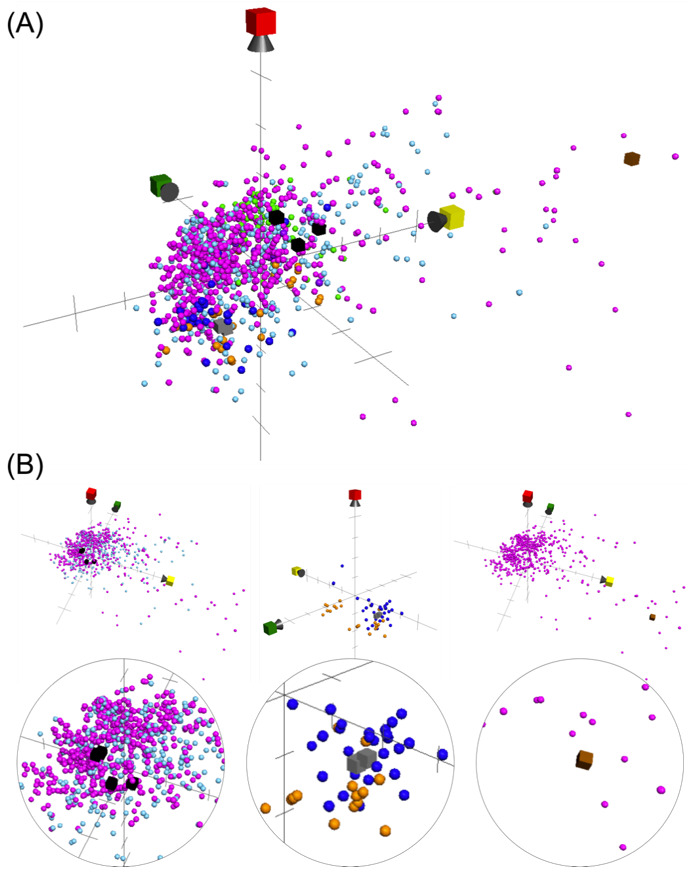
Mode of action (MOA)-relevant chemical patterns plotted by the isolates in the current study [4-methylene steroids (black), nucleosides (grey), and macrolide (brown)], as well as the clinical and preclinical chemotherapeutic agents, including alkylating agents (blue, 31 compounds), anti-metabolites (orange, 24 compounds), proteasome inhibitors (light blue, 457 compounds), tyrosine kinase inhibitors (light green, 102 compounds), topoisomerase (purple, 8 compounds), and anti-microtubule agents (pink-purple, 642 compounds) sorted from the ChEMBL database. The score plot of the three dimensions (principle component) was marked with PC1 (yellow) describing the size, shape, and polarizability; PC2 (green) representing the aromatic- and conjugation-related properties; PC3 (red) depicting lipophilicity, polarity, and H-bond capacity. (**A**) The overall view of analysis; (**B**) the individual view of each type of compound.

To further discover the possible mode of anti-proliferative actions of these compounds, we used a computational tool, ChemGPS-NP (chemical global positioning system for natural products). This method was performed based on the principal component analysis (PCA) of 35 selected physical-chemical properties, including size, shape, polarizability, lipophilicity, polarity, flexibility, rigidity, and hydrogen bond capacity [29]. Chemical structures with similar physical-chemical properties will show in the neighborhood inside the virtual space and can be correlated to similar activity. This system can be applied as a chemo-informatic tool for navigating and charting the biologically relevant chemical space [30]. In our current work, the five major action modes of clinical or preclinical chemotherapeutic agents were selected and sorted from the ChEMBL database. Together with the three types of isolates (4-methylene steroids, nucleosides, and macrolide) isolated in the current study, all compounds were analyzed through a public web interface, ChemGPS-NP_web_ [http://chemgps.bmc.uu.se (accessed on 5 July 2021)] and plotted on the 3D graph to present the physical-chemical relationship between inputted compounds (Figure 5A). The results indicated that the anti-proliferative properties of 4-methylene steroids (labeled as black cubes) may be achieved through blocking the proteasomes actions (proteasome inhibitors, labeled as light blue spheres) as well as stabilizing microtubules to block cell division (anti-microtubule agents, labeled as pink-purple spheres). The isolated nucleosides (labeled as grey cubes) were suggested to cause breakage of the DNA strands by alkylating guanine bases or interfering with the synthetic enzymes, suggesting similar action modes of alkylating agents (labeled as blue spheres) and anti-metabolites (labeled as orange spheres). Due to the large molecular size, our macrolide compound exhibited great value at PC1 (labeled as yellow axis) where only clinical anti-microtubule agents appeared, indicating its possible mechanism of action as blocking cell growth through stopping mitosis.

Gaining an insight into the analyzing results of ChemGPS-NP, the proposed action modes of nucleoside isolates (**5** and **6**) might correlate to the clinical agents such as cytarabine (Ara-C) or vidarabine (Ara-A), which were found to be DNA polymerase inhibitors [31]. Also, the macrolide derivatives have been studied previously to exhibit the anti-cancer properties via disrupting the actin/microtubule cytoskeleton [7,20,21], confirming the anti-microtubule potential of obtained swinholide A (**7**). The further mechanical investigation, aiming to identify the mode of anti-proliferative action of 4-methylene steroids, could focus on the targets of proteasomes and microtubules. Moreover, based on this chemo-informatic approach, we may be able to elucidate the molecular mechanisms more efficiently or to suggest likely chemical functionalities for further anti-cancer medicinal modifications.

## 3. Materials and Methods

### 3.1. General Experimental Procedures

Optical rotation spectra were obtained on a Jasco P-1010 digital polarimeter (Jasco, Tokyo, Japan). UV spectra were recorded using Jasco UV-530 ultraviolet spectrophotometers. IR spectra were detected on the Varian 1000 Scimitar series FT-IR (Varian Inc., Palo Alto, CA, USA). NMR spectroscopic analysis was performed on a 400 or a 500 MHz Varian Mercury Plus FT-NMR (Varian Inc., Palo Alto, CA, USA). HRESIMS data were obtained on a Bruker Daltonics Apex II mass spectrometer (Bruker Daltonics, Bremen, Germany). Silica gel 60, 70–230/230–400 μm ASTM (Merck, Darmstadt, Germany) was used for the normal phase column chromatography. A Hitachi L-7110 pump coupled a Hitachi L-2455 Photodiode Array Detector (Hitachi, Tokyo, Japan) and a column Hibar RT250-10 mm, LiChrosper Si 60, 5 μm (Merck, Darmstadt, Germany) were used for normal phase HPLC chromatography. All methods were conducted following the relevant regulations and guidelines.

### 3.2. Animal Material

The specimen of the wild-type sponge *Theonella* sp. was collected by scuba diving at a depth of about 10 m from the water inlet of a nuclear power plant in Kenting, Pingtung, Taiwan in May 2012. A voucher specimen was deposited at −20 °C at the National Museum of Marine Biology and Aquarium, Taiwan (specimen No. 2012-05-SP). Taxonomic identification was performed by Dr. Bo-Rong Peng, based on the spicule and morphological determination.

### 3.3. Extraction and Isolation

*Theonella* sp. (2 kg fresh weight) was collected and freeze-dried into 500 g dried material. The freeze-dried sponge was minced and extracted three times with ethyl acetate (EtOAc). The extract was dried using a vacuum evaporator to afford a residue (10.1 g), and the residue was subjected to a normal phase column chromatography (silica gel 70–230 mesh) starting with *n*-hexane and the polarity increased with *n*-hexane:EtOAc mixtures, followed by acetone to yield 12 fractions: F1 (eluted by *n*-hexane), F2 (eluted by *n*-hexane:EtOAc, 100:1), F3 (50:1), F4 (20:1), F5 (10:1), F6 (5:1), F7 (3:1), F8 (2:1), F9 (1:1), F10 (1:2), F11 (eluted by EtOAc), and F12 (eluted by acetone). F6 was further separated with a normal phase silica gel column and eluted with (*n*-hexane:EtOAc 10:1 to 5:1) to afford **4** (20.0 mg). F7 was fractionated with normal-phase HPLC with isocratic elution of *n*-hexane:EtOAc 5:1 to yield **2** (10.6 mg). Compounds **1** (1.5 mg) and **3** (8.7 mg) were obtained using normal-phase HPLC with eluting solvent mixtures *n*-hexane:acetone 5:1 and 3:1 to yield F8 and F9, respectively. For the fractions with higher polarity components, F11 was purified using normal-phase HPLC (*n*-hexane:acetone 2:1) to afford **5** (6.5 mg), **6** (0.8 mg), and **7** (16.5 mg). The spectra of NMR and physical data of all compounds were presented at Supplementary Materials (Figures S1–S24).

Theonellasterol L (**1**): Pale-yellow oil; ${\left[\alpha \right]}_{D}^{25}$ +42 (*c* 0.08, CHCl_3_); IR (neat, CHCl_3_) *ν*_max_ 3410 and 1712 cm^−1^; ^1^H and ^13^C NMR data, see Table 1; HRESIMS *m*/*z* 523.3768 [M + Na]^+^ (C_32_H_52_O_4_Na, calculated as 523.3763).

### 3.4. MTT Cell Proliferation Assay

An MTT assay was used to evaluate the cellular proliferation of MCF-7 and MDA-MB-231 (human breast adenocarcinoma), T-47D (human hormone-dependent breast cancer), HCT-116 and DLD-1 (human colon adenocarcinoma), K562 (human chronic myelogenous leukemia), and Molt-4 (human T lymphoblast, acute lymphoblastic leukemia) cancer cell lines after treatment with compounds **1**–**7**. All cell lines were obtained from the American Type Culture Collection (ATCC, Manassas, VA, USA). In brief, cells (1 × 10^5^ cells/mL) were seeded in 96-well plates with 150 μL per well and incubated with several concentrations of compounds **1**–**7** for 24 h. After adding 50 μL of MTT solution (1 mg/mL in PBS), the culture was incubated at 37 °C for 4 h. DMSO (200 μL) was added to dissolve the formazan. The plate was read on an ELISA reader at 595 nm.

### 3.5. ChemGPS-NP Prediction for the Possible Mode of Actions

The ChemGPS-NP [http://chemgps.bmc.uu.se (accessed on 5 July 2021)] is a tool for navigation in biologically relevant chemical space. It has eight principal components (PCs) based on 35 carefully selected chemical descriptors describing physical-chemical properties such as size, shape, polarizability, lipophilicity, polarity, flexibility, rigidity, and hydrogen bond capacity. The ChemGPS-NP prediction scores were calculated for all selected compounds using the online tool ChemGPS-NP_Web_ [http://chemgps.bmc.uu.se (accessed on 5 July 2021)] based on their structural information as a simplified molecular input line entry specification (SMILES) derived via ChemBioDraw version 16.0 (CambridgeSoft, Waltham, MA, USA). All chemotherapeutic agents were sorted from ChEMBL (as keywords of alkylating agents, anti-metabolites, proteasome inhibitors, tyrosine kinase inhibitors, topoisomerase, and anti-microtubule agents), and mapped into the ChemGPS-NP chemical property space using the software Grapher 2.6 (Mac OS, Cupertino, CA, USA).

## 4. Conclusions

The current study focused on characterizing the metabolic diversity and the anti-proliferative potential of the marine sponge *Theonella* sp. and its components. Three classes of compounds were obtained, including 4-methylene steroids (**1**–**4**), nucleosides (**5** and **6**), and macrolide (**7**). Theonellasterol L (**1**) was obtained as a novel compound, while 5′-*O*-acetyl-2′-deoxyuridine (**5**) and 5′-*O*-acetylthymidine (**6**) were found to be the first identified deoxyuridine and thymidine derivatives from the sponge *Theonella* sp. The anti-proliferative assessment and the structure-activity relationship (SAR) analysis pointed out the importance of the 14-/15-oxygenated group among all active 4-methylene steroids. All three classes of the isolated metabolites showed cytotoxic activity, however, the macrolide derivatives were the most potent compounds against most cancer cell lines. ChemGPS-NP analysis was performed to interpret the possible modes of anti-proliferative actions which provided an in-depth analysis of the mechanism of action of the isolated metabolites.

## Figures and Tables

**Table 1:** ^1^H, ^13^C, ^1^H–^1^H COSY, and HMBC NMR data of **1**.

Position	*δ*_H_ (*J* in Hz) ^a^	*δ*_C_ (Mult.) ^b^	^1^H–^1^H COSY	HMBC
1	1.40 m; 1.78 m	36.2 (CH_2_) ^d^	H-2	
2	1.39 m; 2.05 m	32.8 (CH_2_)	H-1, H-3	C-1, C-10
3	4.06 m	73.1 (CH)	H-2	
4		151.9 (C)		
5	2.27 m	43.1 (CH)	H-6	
6	1.68 m; 1.88 m	30.1 (CH_2_)	H-5, H-7	
7	5.93 br s	70.8 (CH)	H-6	
8		129.8 (C)		
9	2.32 t (8.5) ^c^	44.6 (CH)	H-11	
10		40.2 (C)		
11	1.53 m; 1.72 m	19.9 (CH_2_)	H-9, H-12	
12	1.30 m; 1.98 m	36.7 (CH_2_)	H-11	
13		43.8 (C)		
14		154.1 (C)		
15	4.80 d (6.0)	69.2 (CH)	H-16	
16	1.72 m; 1.86 m	38.6 (CH_2_)	H-15, H-17	
17	1.58 m	53.0 (CH)	H-16, H-20	
18	0.84 s	18.9 (CH_3_)		C-12, C-13, C-14, C-17
19	0.65 s	12.8 (CH_3_)		C-1, C-5, C-9, C-10
20	1.42 m	34.1 (CH)	H-17, H-21, H-22	
21	0.96 d (6.5)	19.2 (CH_3_)	H-20	C-17, C-20, C-22
22	1.02 m; 1.42 m	33.8 (CH_2_)	H-20, H-23	
23	1.04 m; 1.34 m	26.6 (CH_2_)	H-22, H-24	
24	0.94 m	46.1 (CH)	H-23, H-25, H-28	
25	1.68 m	28.9 (CH)	H-24, H-26, H-27	
26	0.82 d (7.0)	18.9 (CH_3_)	H-25	C-24, C-25
27	0.83 d (6.5)	19.6 (CH_3_)	H-25	C-24, C-25
28	1.14 m; 1.32 m	22.9 (CH_2_)	H-24, H-29	C-23, C-24, C-25
29	0.86 t (7.5)	12.3 (CH_3_)	H-28	C-24
30	4.60 s; 5.12 s	103.3 (CH_2_)		C-3, C-4, C-5
7-OAc	2.04 s	21.8 (CH_3_)		
		171.2 (C)		

^a^ 500 MHz in CDCl_3_; ^b^ 125 MHz in CDCl_3_; ^c^ *J* values (Hz) are given in parentheses; ^d^ numbers of the attached protons were deduced by DEPT experiments.

**Table 2:** Anti-proliferative activity of the isolated metabolites from the sponge *Theonella* sp.

Compounds	Cell Lines (IC_50_ μM)						
	MCF-7	MDA-MB-231	t-47D	HCT-116	DLD-1	K562	Molt4
**1**	– ^a^	– ^a^	– ^a^	– ^a^	– ^a^	17.60	18.06
**2**	– ^a^	– ^a^	– ^a^	– ^a^	– ^a^	– ^a^	– ^a^
**3**	– ^a^	– ^a^	– ^a^	13.36	– ^a^	7.87	14.14
**4**	– ^a^	– ^a^	– ^a^	– ^a^	– ^a^	– ^a^	– ^a^
**5**	– ^a^	– ^a^	18.08	– ^a^	2.50	10.93	8.61
**6**	– ^a^	5.98	– ^a^	– ^a^	– ^a^	– ^a^	– ^a^
**7**	1.03	– ^a^	0.65	0.78	0.55	0.01	0.02
Doxorubicin ^b^	1.10	1.16	0.61	0.27	0.72	0.62	0.04

^a^ IC_50_ > 20 μM; ^b^ positive control.

## Data Availability

The data presented in this study are available within the article and Supplementary Materials.

## Supplementary Materials

The following data are available online at https://www.mdpi.com/article/10.3390/metabo11080532/s1, Figure S1: IR spectrum of **1**, Figure S2: ESIMS spectrum of **1**, Figure S3: HRESIMS spectrum of **1**, Figure S4: ^1^H NMR (500 MHz, CDCl_3_) spectrum of **1**, Figure S5: ^1^H NMR (500 MHz, CDCl_3_) spectrum of **1** (Partial enlarged view), Figure S6: 1H NMR (500 MHz, CDCl_3_) spectrum of **1** (Partial enlarged view), Figure S7: ^13^C NMR (125 MHz, CDCl_3_) spectrum of **1**, Figure S8: DEPT spectrum of **1**, Figure S9: HSQC spectrum of **1**, Figure S10: HMBC spectrum of **1**, Figure S11: COSY spectrum of **1**, Figure S12: NOESY spectrum of **1**, Figure S13: ^1^H NMR (500 MHz, CDCl_3_) spectrum of **2**, Figure S14: ^13^C NMR (125 MHz, CDCl_3_) spectrum of **2**, Figure S15: ^1^H NMR (500 MHz, CDCl_3_) spectrum of **3**, Figure S16: ^13^C NMR (125 MHz, CDCl_3_) spectrum of **3**, Figure S17: ^1^H NMR (500 MHz, CDCl_3_) spectrum of **4**, Figure S18: ^13^C NMR (125 MHz, CDCl_3_) spectrum of **4**, Figure S19: ^1^H NMR (500 MHz, CDCl_3_) spectrum of **5**, Figure S20: ^13^C NMR (125 MHz, CDCl_3_) spectrum of **5**, Figure S21: ^1^H NMR (500 MHz, CDCl_3_) spectrum of **6**, Figure S22: ^13^C NMR (125 MHz, CDCl_3_) spectrum of **6**, Figure S23: ^1^H NMR (500 MHz, CDCl_3_) spectrum of **7**, Figure S24: ^13^C NMR (125 MHz, CDCl_3_) spectrum of **7**.

## References

[1] Dahiya R., Dahiya S., Kumar P., Kumar R.V., Dahiya S., Kumar S., Saharan R., Basu P., Mitra A., Sharma A. (2021). Structural and biological aspects of natural bridged macrobicyclic peptides from marine resources. Arch. Der Pharm..

[2] Peng B.-R., Lai K.-H., Chen Y.-Y., Su J.-H., Huang Y.M., Chen Y.-H., Lu M.-C., Yu S.S.-F., Duh C.-Y., Sung P.-J. (2020). Probing anti-proliferative 24-homoscalaranes from a sponge *Lendenfeldia* sp.. Mar. Drugs.

[3] Tran A.A., Miljkovic M., Prasad V. (2020). Analysis of estimated clinical benefit of newly approved drugs for US patients with acute myeloid leukemia. Leuk. Res..

[4] Bewley C.A., Faulkner D.J. (1998). Lithistid sponges: Star performers or hosts to the stars. Angew. Chem. Int. Ed..

[5] Guo J.-K., Chiang C.-Y., Lu M.-C., Chang W.-B., Su J.-H. (2012). 4-Methylenesterols from a sponge *Theonella swinhoei*. Mar. Drugs.

[6] Ichiba T., Nakao Y., Scheuer P.J., Sata N.U., Kelly-Borges M. (1995). Kumusine, a chloroadenine riboside from a sponge, *Theonella* sp.. Tetrahedron Lett..

[7] Humisto A., Jokela J., Liu L., Wahlsten M., Wang H., Permi P., Machado J.P., Antunes A., Fewer D.P., Sivonen K. (2018). The swinholide biosynthesis gene cluster from a terrestrial cyanobacterium, *Nostoc* sp. strain UHCC 0450. Appl. Environ. Microbiol..

[8] Sinisi A., Calcinai B., Cerrano C., Dien H.A., Zampella A., D’Amore C., Renga B., Fiorucci S., Taglialatela-Scafati O. (2013). Isoswinholide B and swinholide K, potently cytotoxic dimeric macrolides from *Theonella swinhoei*. Bioorg. Med. Chem..

[9] De Marino S., Festa C., D’Auria M.V., Cresteil T., Debitus C., Zampella A. (2011). Swinholide J, a potent cytotoxin from the marine sponge *Theonella swinhoei*. Mar. Drugs.

[10] Cornelio K., Espiritu R., Hanashima S., Todokoro Y., Malabed R., Kinoshita M., Matsumori N., Murata M., Nishimura S., Kakeya H. (2019). Theonellamide A, a marine-sponge-derived bicyclic peptide, binds to cholesterol in aqueous DMSO: Solution NMR-based analysis of peptide-sterol interactions using hydroxylated sterol. Biochim. Biophys. Acta Biomembr..

[11] Fukuhara K., Takada K., Okada S., Matsunaga S. (2016). Nazumazoles D-F, cyclic pentapeptides that inhibit chymotrypsin, from the marine sponge *Theonella swinhoei*. J. Nat. Prod..

[12] Fukuhara K., Takada K., Okada S., Matsunaga S. (2015). Nazumazoles A-C, cyclic pentapeptides dimerized through a disulfide bond from the marine sponge *Theonella swinhoei*. Org. Lett..

[13] Kuranaga T., Enomoto A., Tan H., Fujita K., Wakimoto T. (2017). Total synthesis of theonellapeptolide Id. Org. Lett..

[14] Ratnayake A.S., Bugni T.S., Feng X., Harper M.K., Skalicky J.J., Mohammed K.A., Andjelic C.D., Barrows L.R., Ireland C.M. (2006). Theopapuamide, a cyclic depsipeptide from a Papua New Guinea lithistid sponge *Theonella swinhoei*. J. Nat. Prod..

[15] Fusetani N., Warabi K., Nogata Y., Nakao Y., Matsunaga S., van Soest R.R.M. (1999). Koshikamide A1, a new cytotoxic linear peptide isolated from a marine sponge, *Theonella* sp.. Tetrahedron Lett..

[16] Barbosa M.C.S., Barbosa C.D.S., Oliveira J.T., Moreira N.C.D.S., Martins N.R.D.M., Gomes G.K.A., Caldeira C.A., e Costa M.L.A., Guimarães D.S.M., Guimaraes L. (2018). Synthesis and evaluation of the mutagenicity of 3-alkylpyridine marine alkaloid analogues with anticancer potential. Mutat. Res. Genet. Toxicol. Environ. Mutagen..

[17] Rodriguez J., Jimenez C., Blanco M., Tarazona G., Fernandez R., Cuevas C. (2016). Lanesoic acid: A cytotoxic zwitterion from *Theonella* sp.. Org. Lett..

[18] Yang F., Li Y.Y., Tang J., Sun F., Lin H.W. (2018). New 4-methylidene sterols from the marine sponge *Theonella swinhoei*. Fitoterapia.

[19] Jimenez P.C., Wilke D.V., Branco P.C., Bauermeister A., Rezende-Teixeira P., Gaudencio S.P., Costa-Lotufo L.V. (2020). Enriching cancer pharmacology with drugs of marine origin. Br. J. Pharmacol..

[20] Youssef D.T., Mooberry S.L. (2006). Hurghadolide A and swinholide I, potent actin-microfilament disrupters from the Red Sea sponge *Theonella swinhoei*. J. Nat. Prod..

[21] Bubb M.R., Spector I., Bershadsky A.D., Korn E.D. (1995). Swinholide A is a microfilament disrupting marine toxin that stabilizes actin dimers and severs actin filaments. J. Biol. Chem..

[22] De Marino S., Ummarino R., D’Auria M.V., Chini M.G., Bifulco G., Renga B., D’Amore C., Fiorucci S., Debitus C.C., Zampella A. (2011). Theonellasterols and conicasterols from *Theonella swinhoei*—Novel marine natural ligands for human nuclear receptors. J. Med. Chem..

[23] Kho E., Imagawa D.K., Rohmer M., Kashman Y., Djerassi C. (2002). Sterols in marine invertebrates. 22. Isolation and structure elucidation of conicasterol and theonellasterol, two new 4-methylene sterols from the Red Sea sponges *Theonella conica* and *Theonella swinhoei*. J. Org. Chem..

[24] Nguyen C., Kasinathan G., Leal-Cortijo I., Musso-Buendia A., Kaiser M., Brun R., Ruiz-Pérez L.M., Johansson N.G., González-Pacanowska D., Gilbert I.H. (2005). Deoxyuridine triphosphate nucleotidohydrolase as a potential antiparasitic drug target. J. Med. Chem..

[25] Ahmed A.F., Wu M.-H., Wu Y.-C., Dai C.-F., Sheu J.-H. (2006). Metabolites with cytotoxic activity from the formosan soft coral *Cladiella australis*. J. Chin. Chem. Soc..

[26] Doi M., Ishida T., Kobayashi M., Kitagawa I. (2002). Molecular conformation of swinholide A, a potent cytotoxic dimeric macrolide from the Okinawan marine sponge *Theonella swinhoei*: X-ray crystal structure of its diketone derivative. J. Org. Chem..

[27] Madaio A., Notaro G., Piccialli V., Sica D. (2004). Minor 5,6-secosterols from the marine sponge *Hippospongia communis*—Solation and synthesis of (7Z,22E,24R)-24-methyl-5,6-secocholesta-7,22-diene-3β,5β,6-triol. J. Nat. Prod..

[28] Qureshi A., Faulkner D.J. (2000). 7α-Hydroxytheonellasterol, a cytotoxic 4-methylene sterol from the Philippines sponge *Theonella swinhoei*. J. Nat. Prod..

[29] Larsson J., Gottfries J., Muresan S., Backlund A. (2007). ChemGPS-NP: Tuned for navigation in biologically relevant chemical space. J. Nat. Prod..

[30] Rosen J., Lovgren A., Kogej T., Muresan S., Gottfries J., Backlund A. (2009). ChemGPS-NP_(Web)_: Chemical space navigation online. J. Comput. Aided Mol. Des..

[31] Iliakis G., Bryant P.E. (1983). Effects of the nucleoside analogues alpha-ara A, beta-ara A and beta-ara C on cell growth and repair of both potentially lethal damage and DNA double strand breaks in mammalian cells in culture. Anticancer Res..

