# Supplementary material for: Anti-Proliferative Potential of Secondary Metabolites from the Marine Sponge Theonella sp.: Moving from Correlation toward Causation
Source: Metabolites. 2021 Aug 10;11(8):532. doi: 10.3390/metabo11080532 (PMC8400523; doi:10.3390/metabo11080532)
Supplement: Supplementary file 1 [file metabolites-11-00532-s001.zip › metabolites-1316684-supplementary.pdf]

SUPPORTING INFORMATION

Article

Anti-Proliferative Potential of Secondary Metabolites from the Marine Sponge *Theonella* sp.: Moving from Correlation toward Causation

Kuei-Hung Lai [1,2,3,*], Bo-Rong Peng [4,5], Chun-Han Su [6], Mohamed El-Shazly [7,8], Yi-Long Sun [9], Ming-Cheng Shih [9], Yu-Ting Huang [10], Pei-Tzu Yen [11], Lung-Shuo Wang [11,12,*] and Jui-Hsin Su [4,5,*]

Table of Contents

Figure S1. IR spectrum of **1** ---------- 2
Figure S2. ESIMS spectrum of **1** ---------- 2
Figure S3. HRESIMS spectrum of **1** ---------- 3
Figure S4. ^{1}H NMR (500 MHz, $CDCl_3$) spectrum of **1** ---------- 3
Figure S5. ^{1}H NMR (500 MHz, $CDCl_3$) spectrum of **1** (Partial enlarged view) ----------4
Figure S6. ^{1}H NMR (500 MHz, $CDCl_3$) spectrum of **1** (Partial enlarged view) ----------4
Figure S7. ^{13}C NMR (125 MHz, $CDCl_3$) spectrum of **1** ---------- 5
Figure S8. DEPT spectrum of **1** ---------- 5
Figure S9. HSQC spectrum of **1** ---------- 6
Figure S10. HMBC spectrum of **1** ---------- 6
Figure S11. COSY spectrum of **1** ---------- 7
Figure S12. NOESY spectrum of **1** ---------- 7
Figure S13. ^{1}H NMR (500 MHz, $CDCl_3$) spectrum of **2** ---------- 8
Figure S14. ^{13}C NMR (125 MHz, $CDCl_3$) spectrum of **2** ---------- 8
Figure S15. ^{1}H NMR (500 MHz, $CDCl_3$) spectrum of **3** ---------- 9
Figure S16. ^{13}C NMR (125 MHz, $CDCl_3$) spectrum of **3** ---------- 9
Figure S17. ^{1}H NMR (500 MHz, $CDCl_3$) spectrum of **4** ---------- 10
Figure S18. ^{13}C NMR (125 MHz, $CDCl_3$) spectrum of **4** ---------- 10
Figure S19. ^{1}H NMR (500 MHz, $CDCl_3$) spectrum of **5** ---------- 11
Figure S20. ^{13}C NMR (125 MHz, $CDCl_3$) spectrum of **5** ---------- 11
Figure S21. ^{1}H NMR (500 MHz, $CDCl_3$) spectrum of **6** ---------- 12
Figure S22. ^{13}C NMR (125 MHz, $CDCl_3$) spectrum of **6** ---------- 12
Figure S23. ^{1}H NMR (500 MHz, $CDCl_3$) spectrum of **7** ---------- 13
Figure S24. ^{13}C NMR (125 MHz, $CDCl_3$) spectrum of **7** ---------- 13

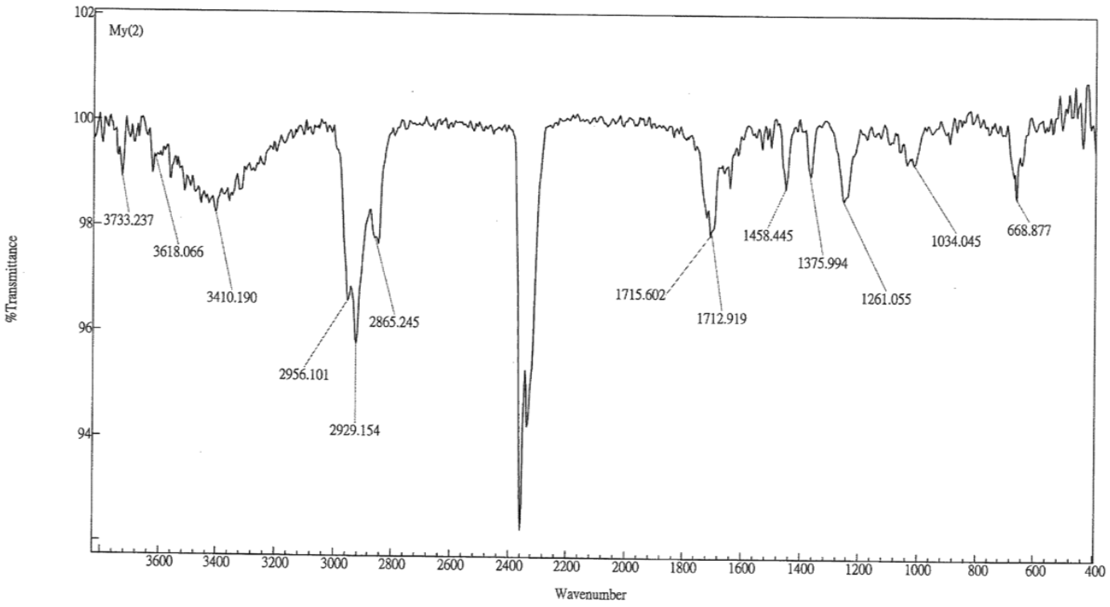

Figure S1. IR spectrum of **1**

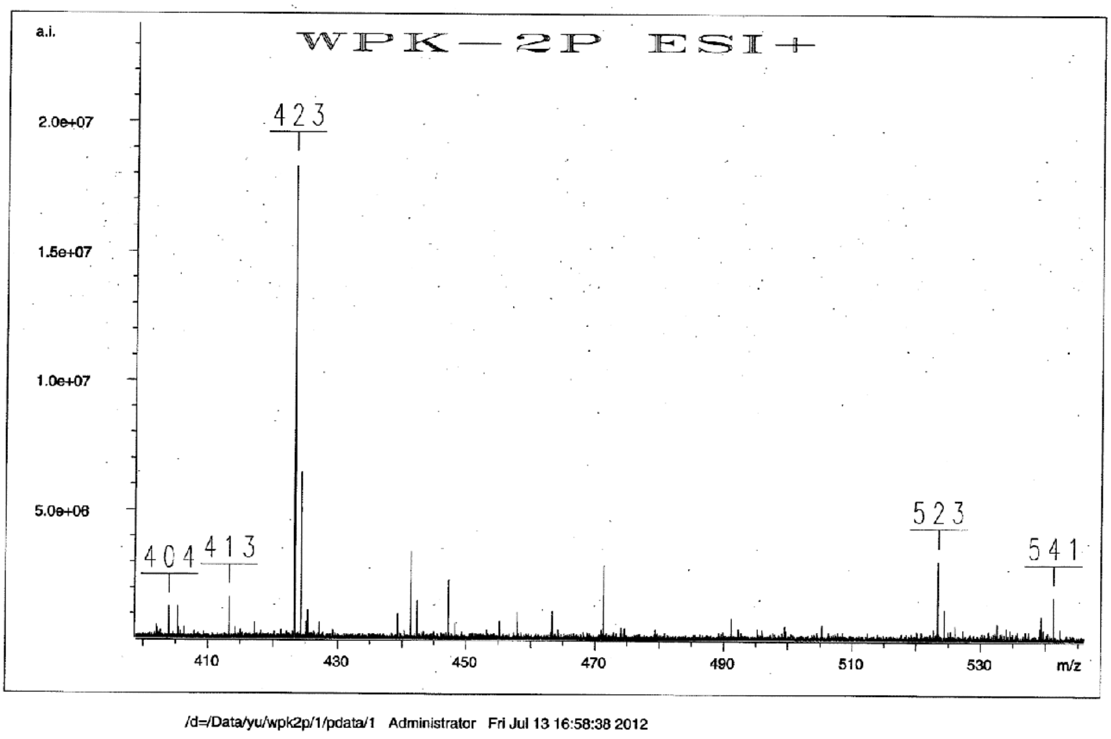

Figure S2. ESIMS spectrum of **1**

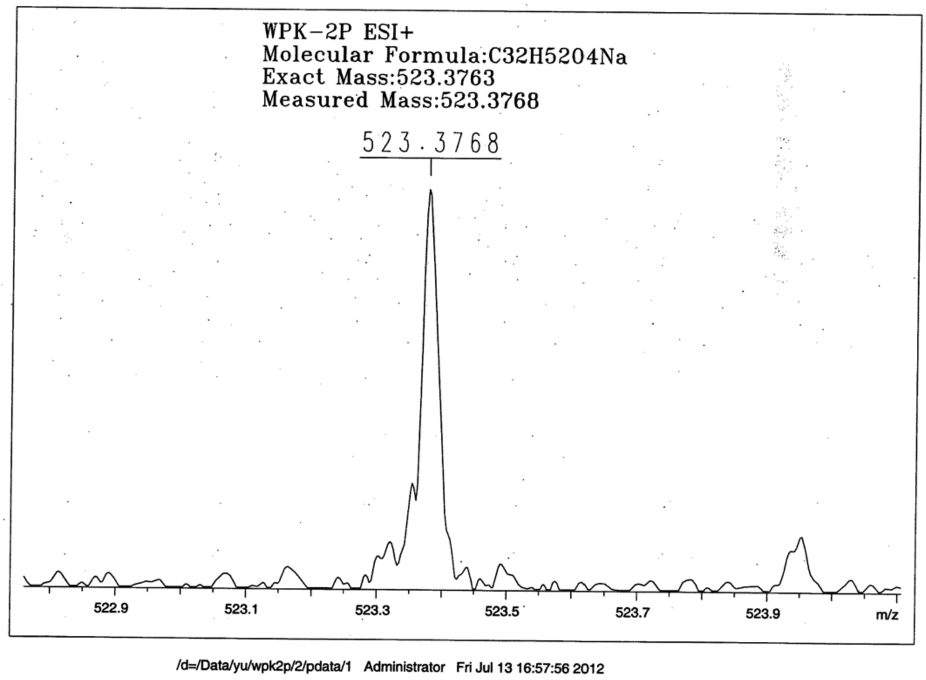

Figure S3. HRESIMS spectrum of **1**

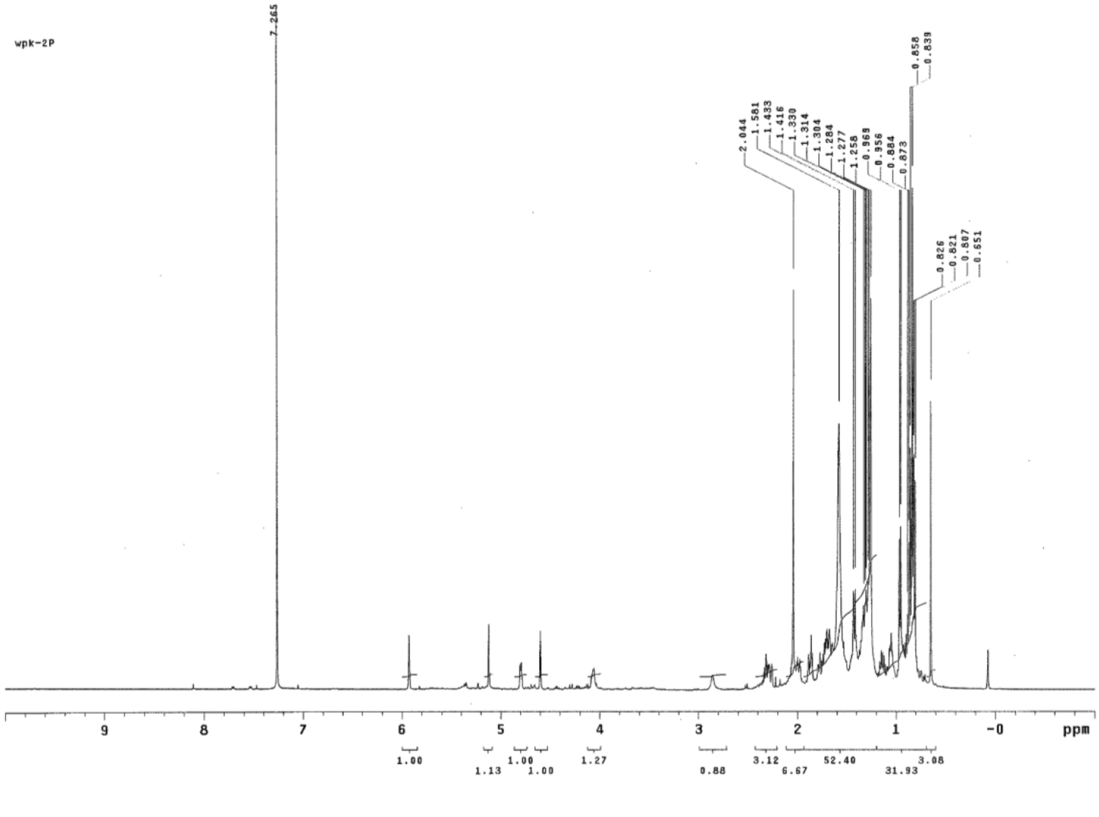

Figure S4. ^{1}H NMR (500 MHz, $CDCl_3$) spectrum of **1**

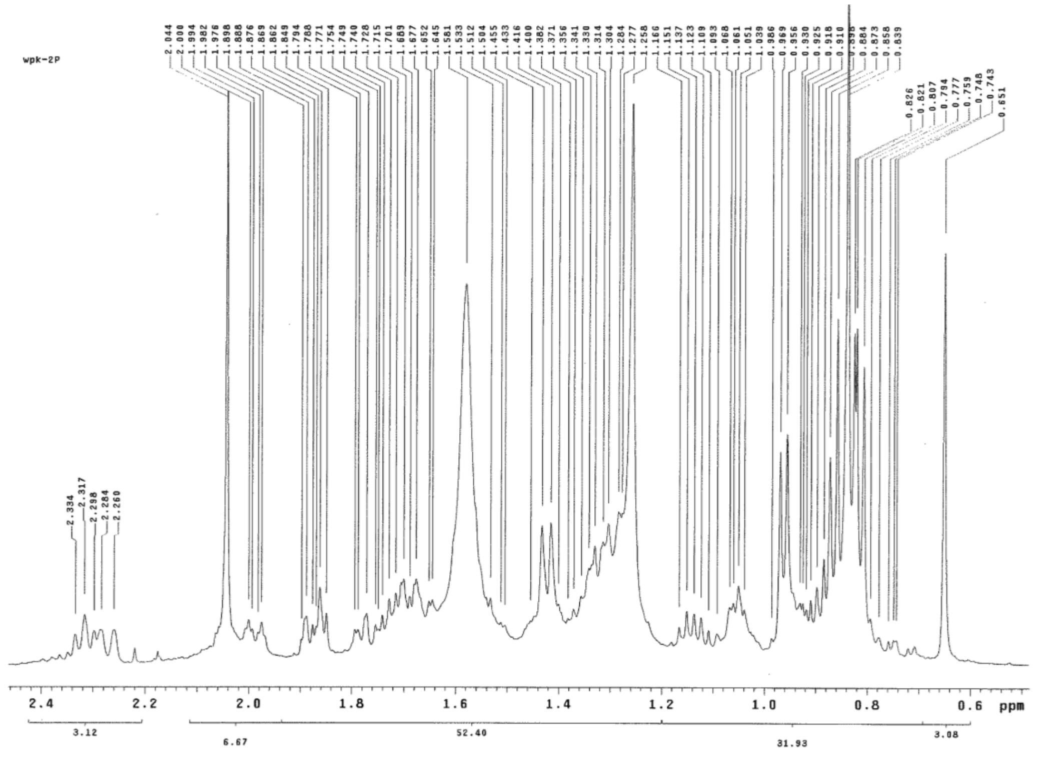

Figure S5. ^{1}H NMR (500 MHz, $CDCl_3$) spectrum of **1** (Partial enlarged view)

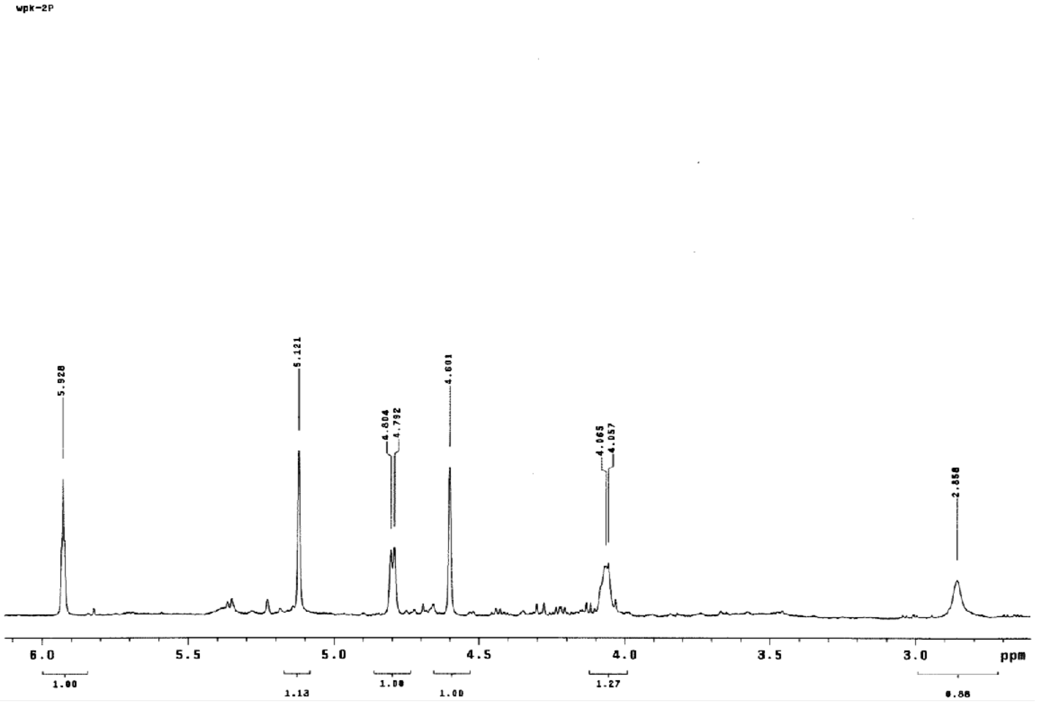

Figure S6. ^{1}H NMR (500 MHz, $CDCl_3$) spectrum of **1** (Partial enlarged view)

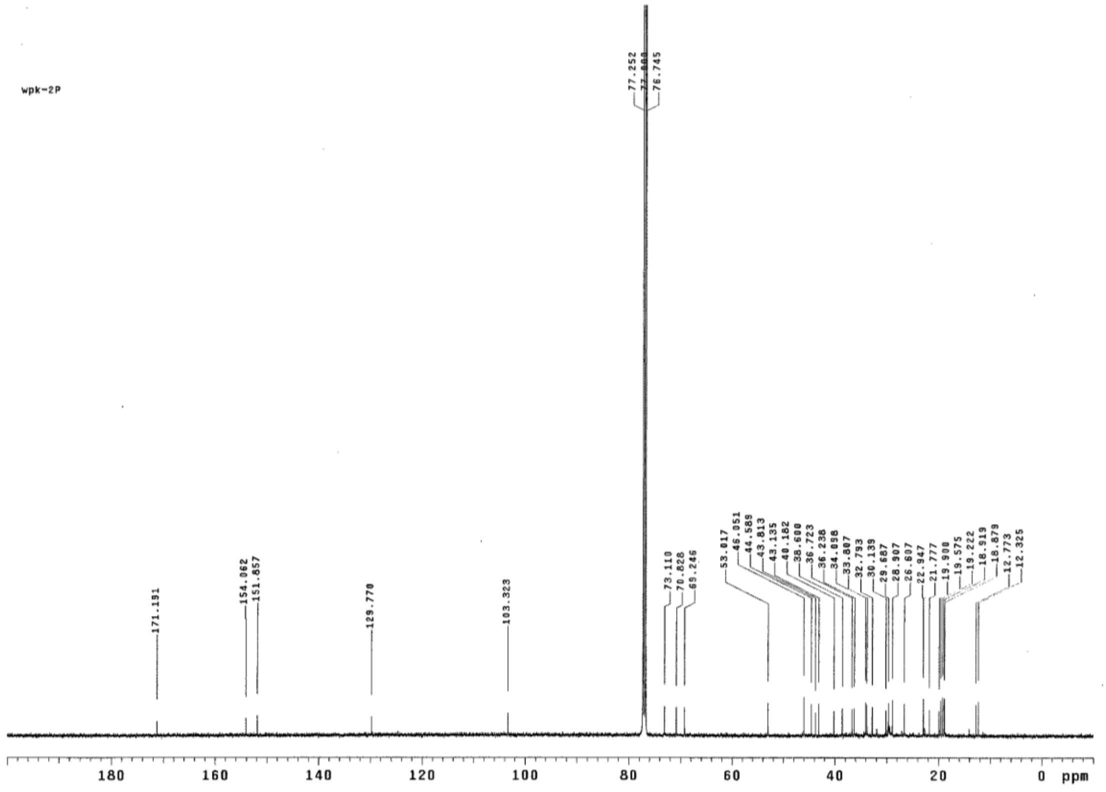

Figure S7. ^{13}C NMR (125 MHz, $CDCl_3$) spectrum of **1**

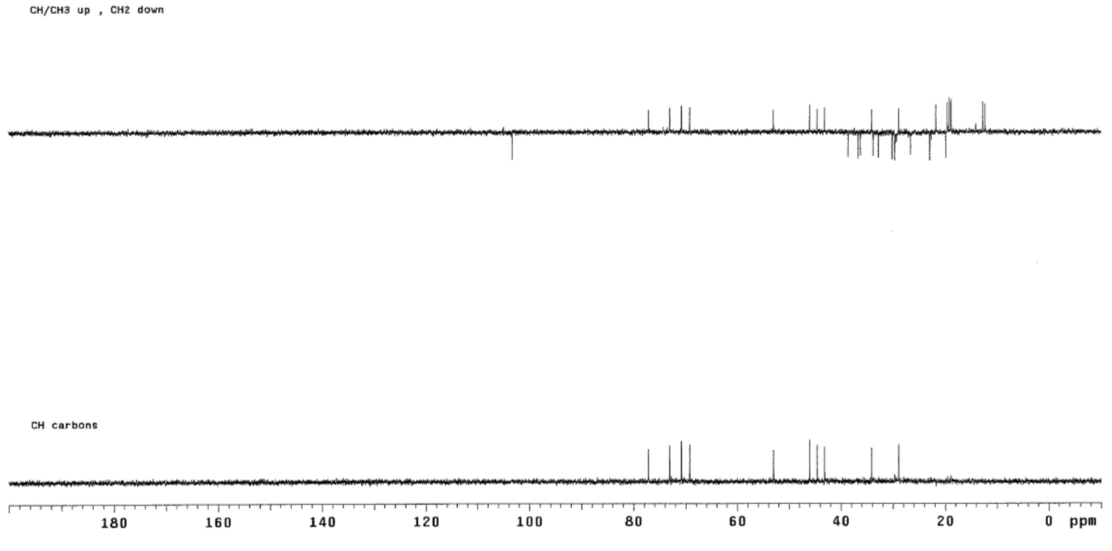

Figure S8. DEPT spectrum of **1**

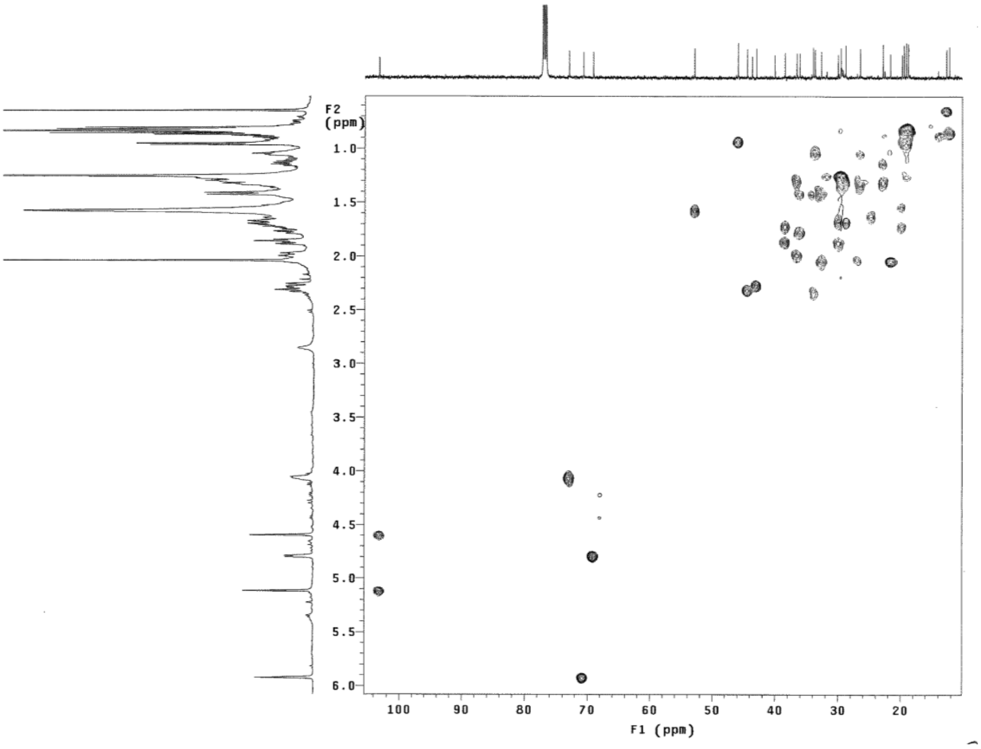

Figure S9. HSQC spectrum of **1**

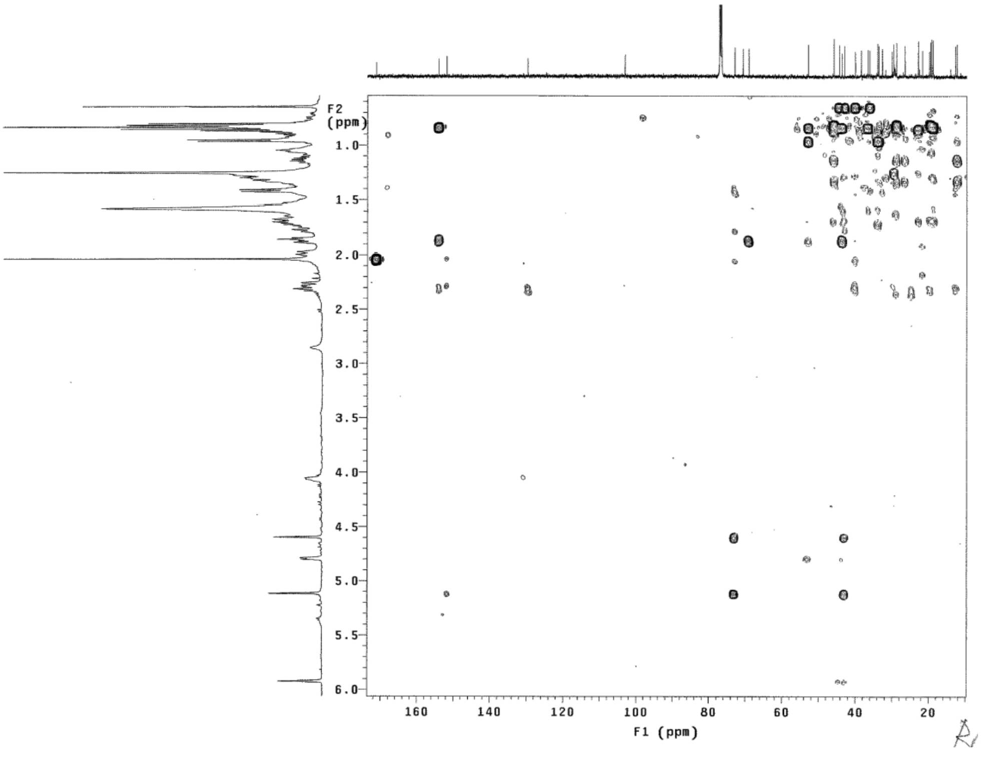

Figure S10. HMBC spectrum of **1**

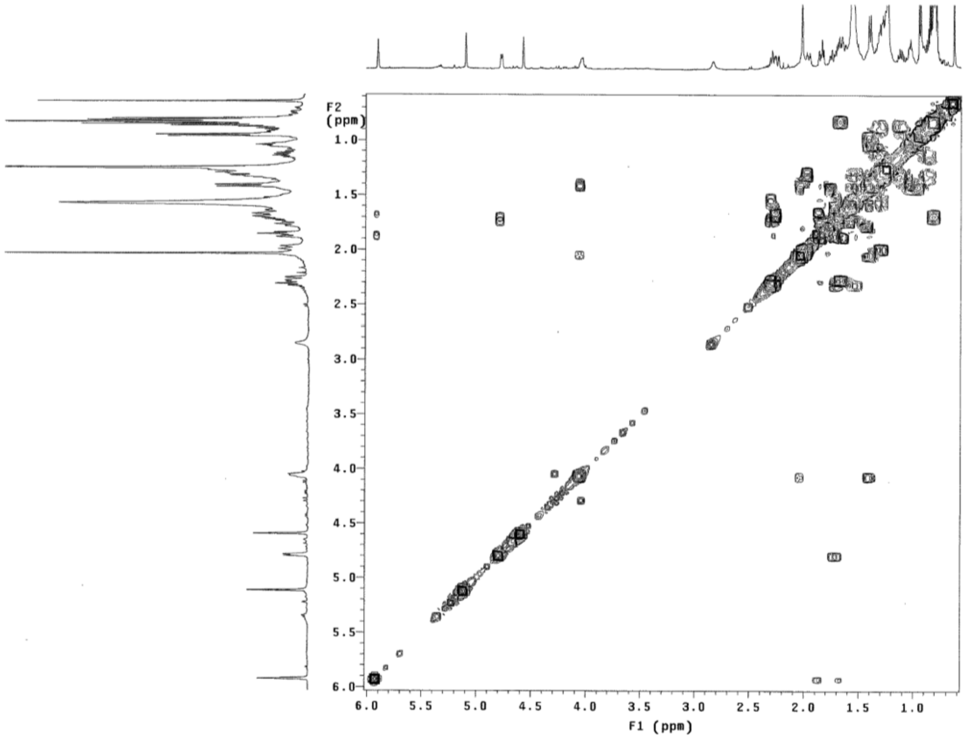

Figure S11. COSY spectrum of **1**

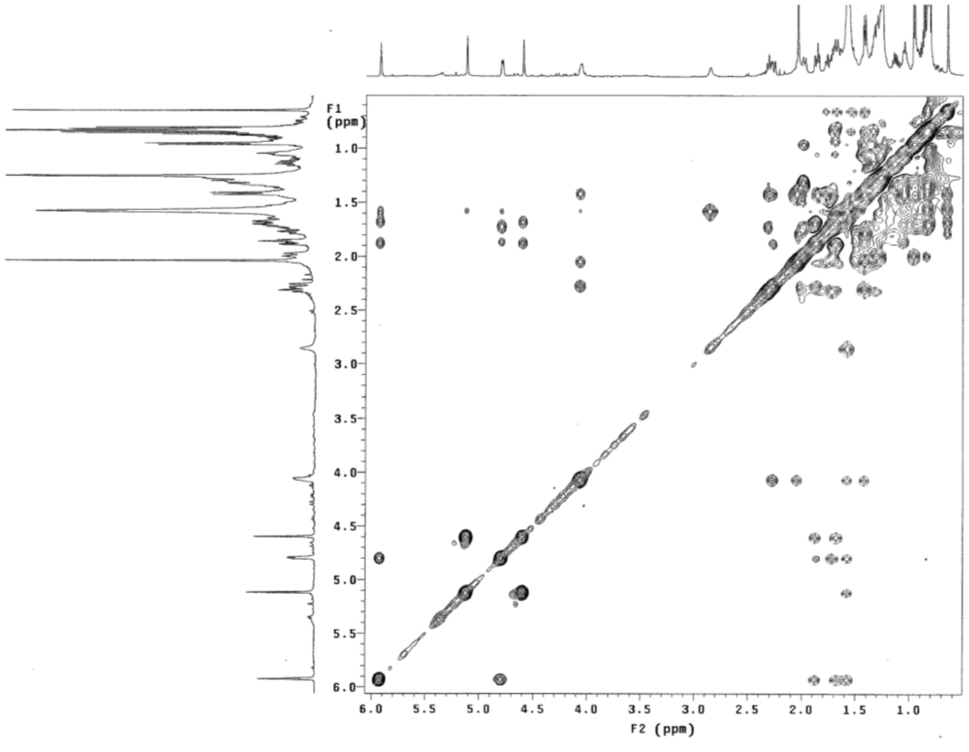

Figure S12. NOESY spectrum of **1**

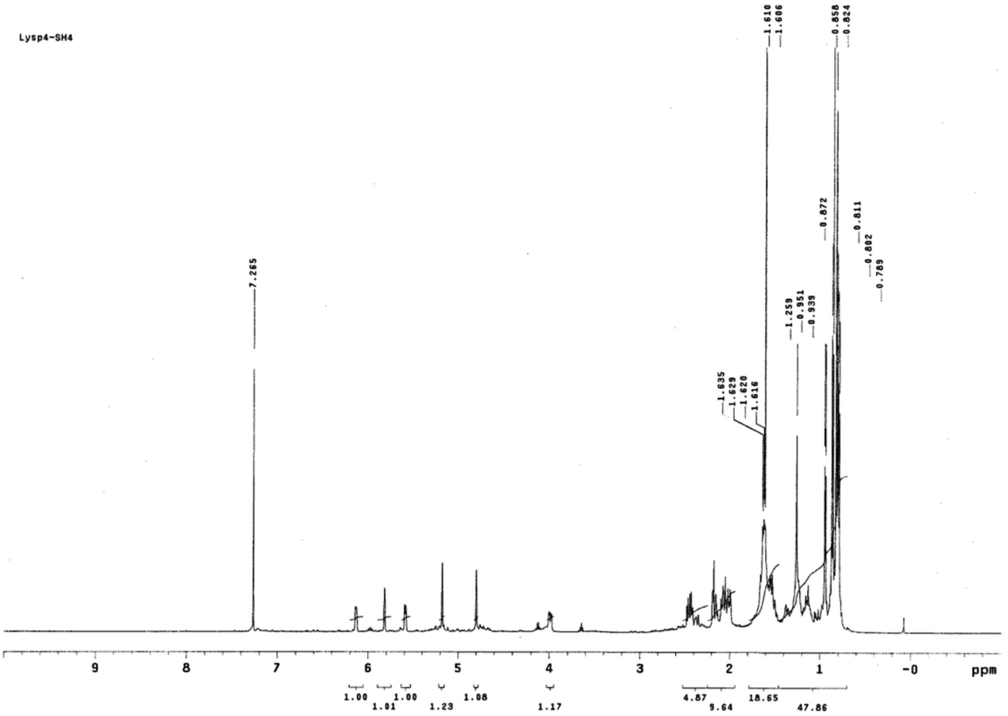

Figure S13. ^{1}H NMR (500 MHz, $CDCl_3$) spectrum of **2**

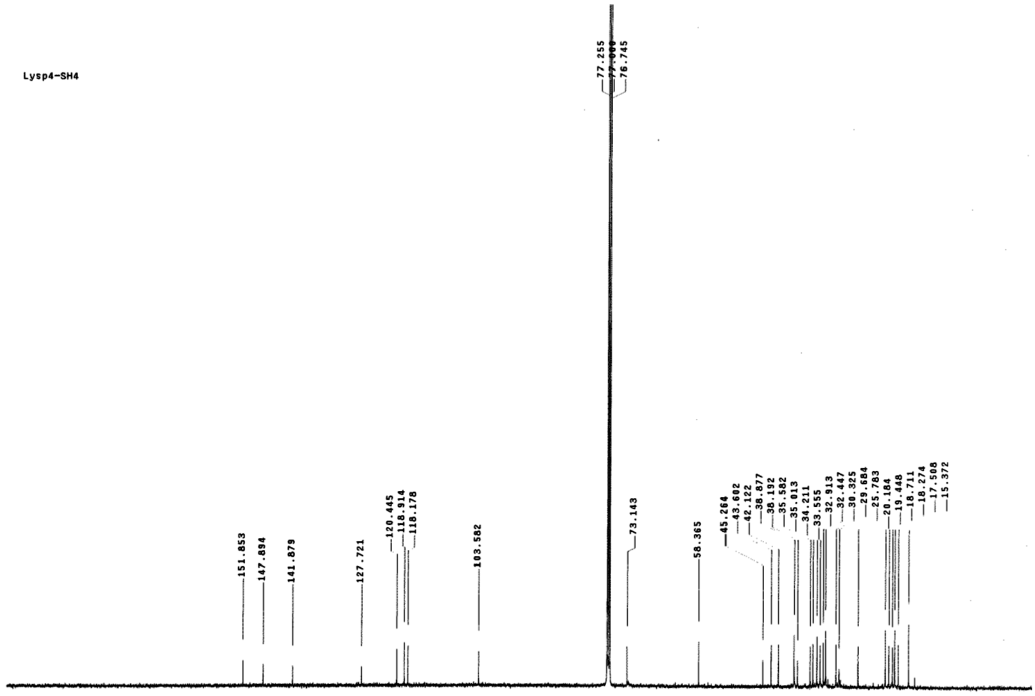

Figure S14. ^{13}C NMR (125 MHz, $CDCl_3$) spectrum of **2**

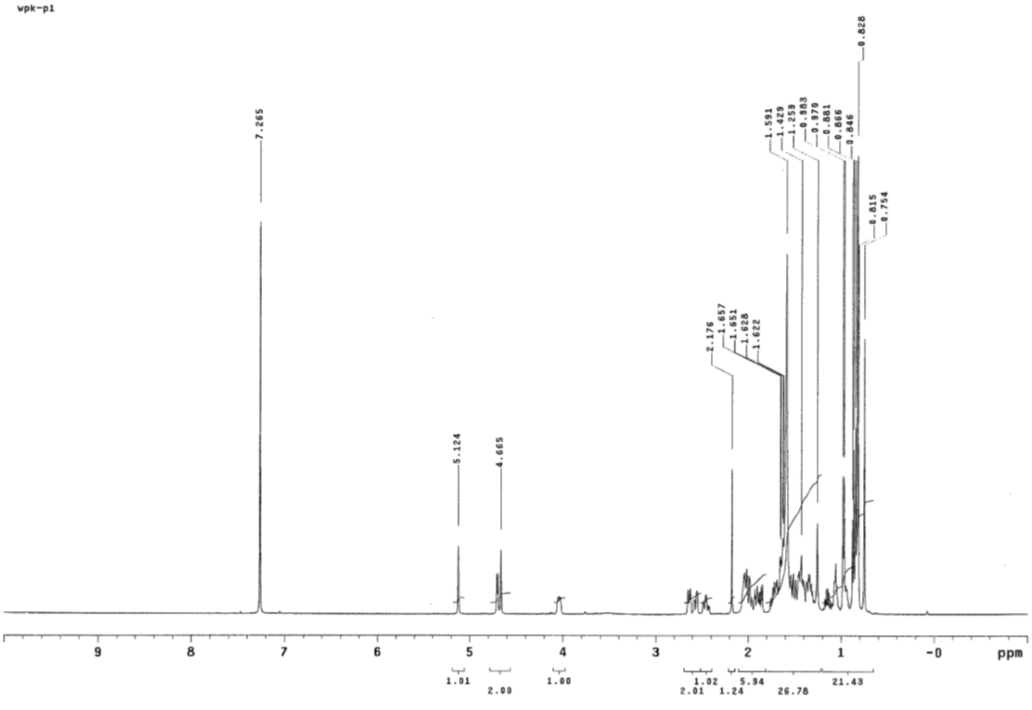

Figure S15. ^{1}H NMR (500 MHz, $CDCl_3$) spectrum of **3**

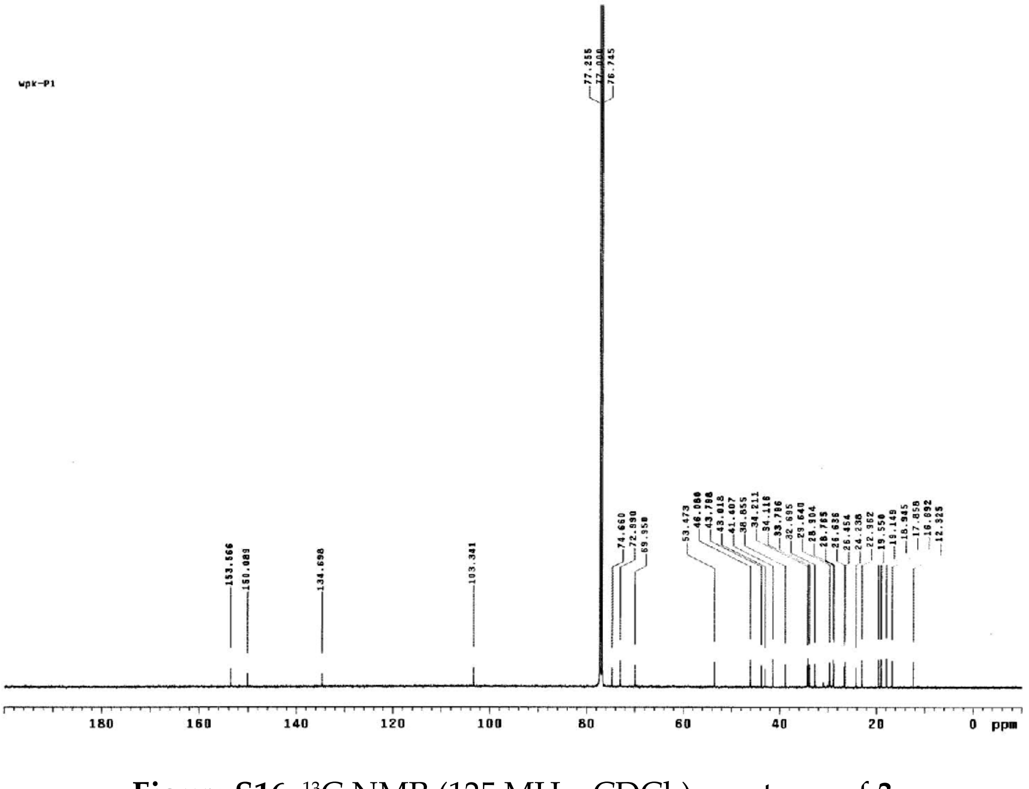

Figure S16. ^{13}C NMR (125 MHz, $CDCl_3$) spectrum of **3**

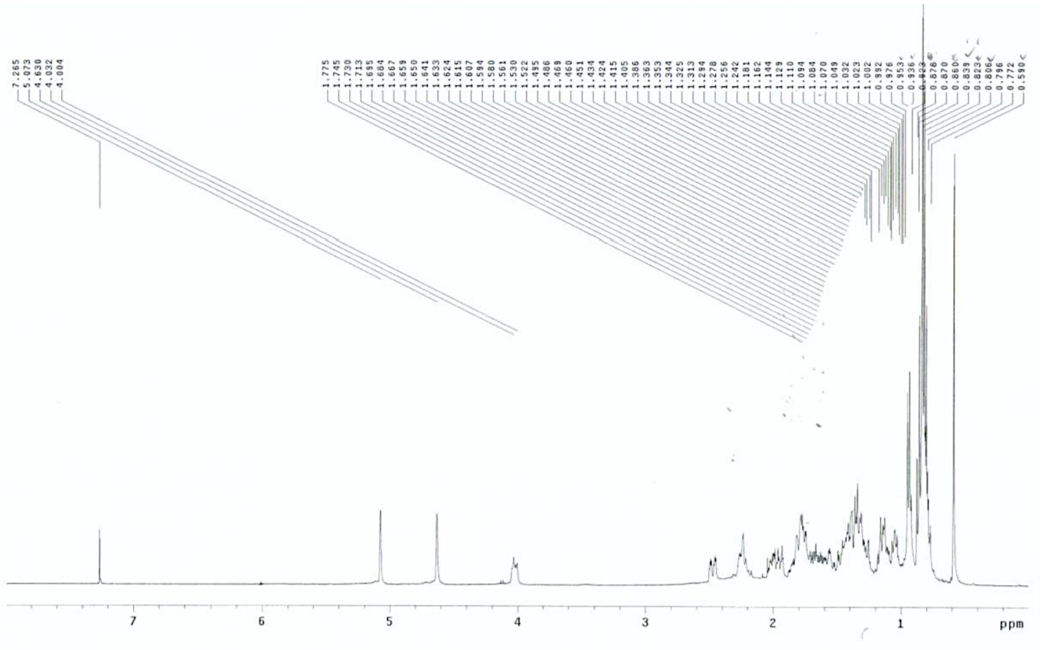

Figure S17. ^{1}H NMR (500 MHz, $CDCl_3$) spectrum of **4**

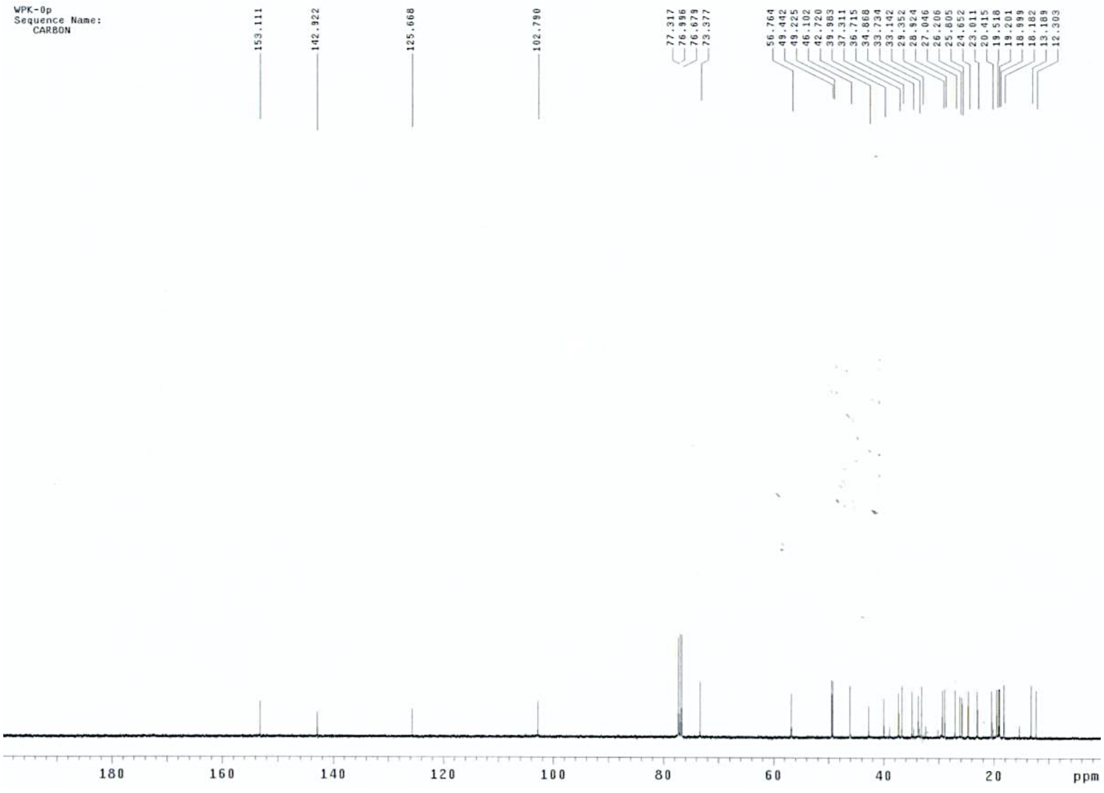

Figure S18. ^{13}C NMR (125 MHz, $CDCl_3$) spectrum of **4**

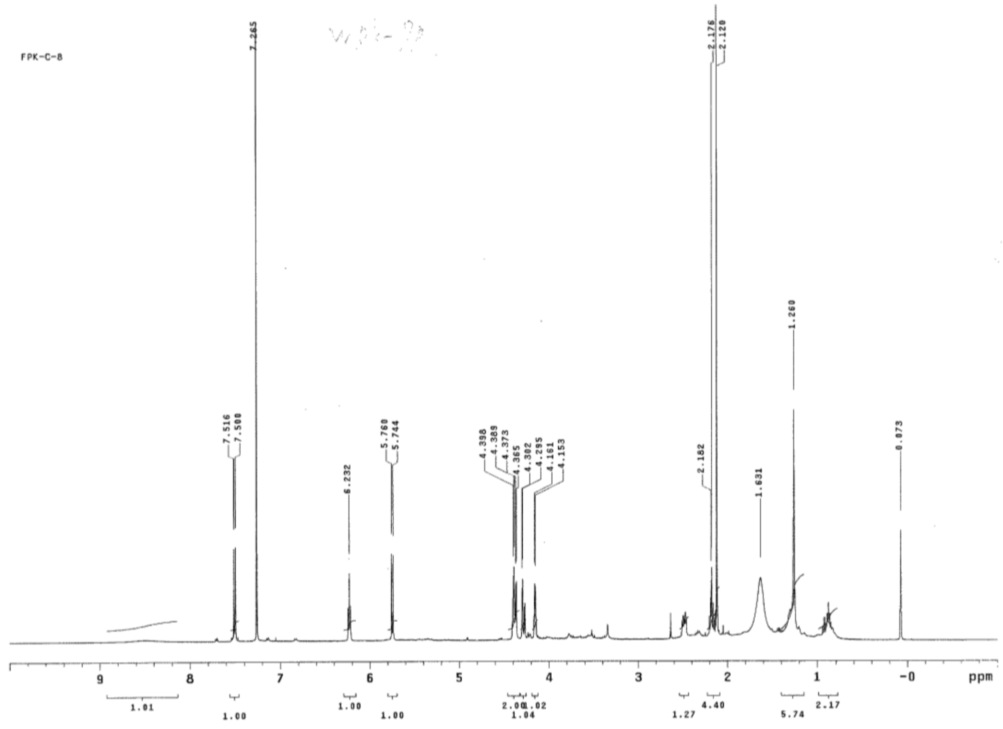

Figure S19. ^{1}H NMR (500 MHz, $CDCl_3$) spectrum of **5**

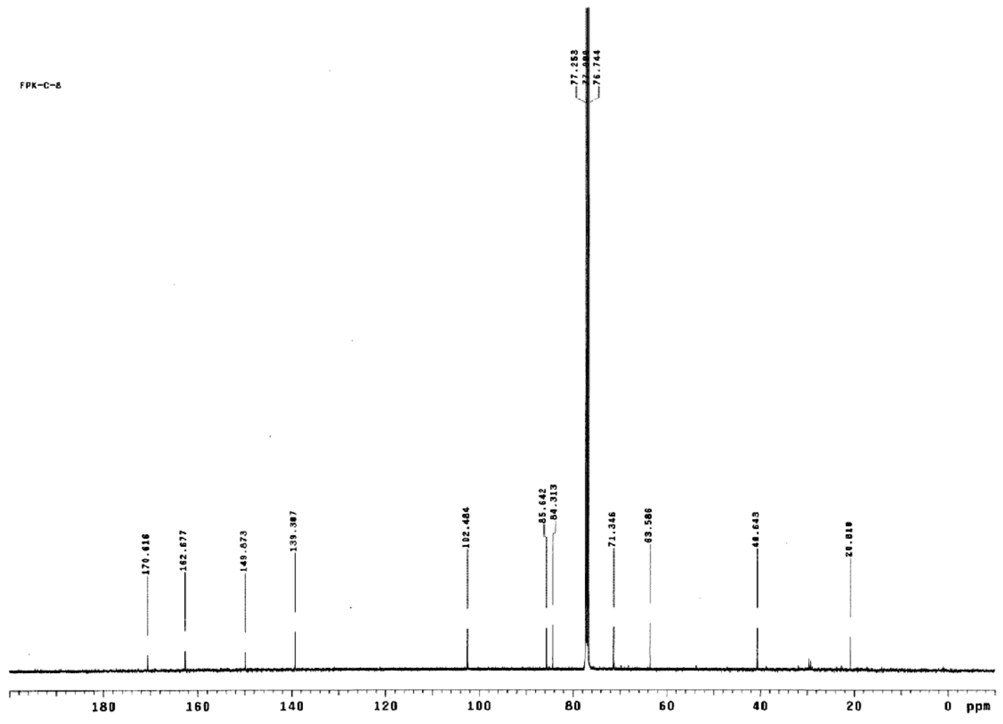

Figure S20. ^{13}C NMR (125 MHz, $CDCl_3$) spectrum of **5**

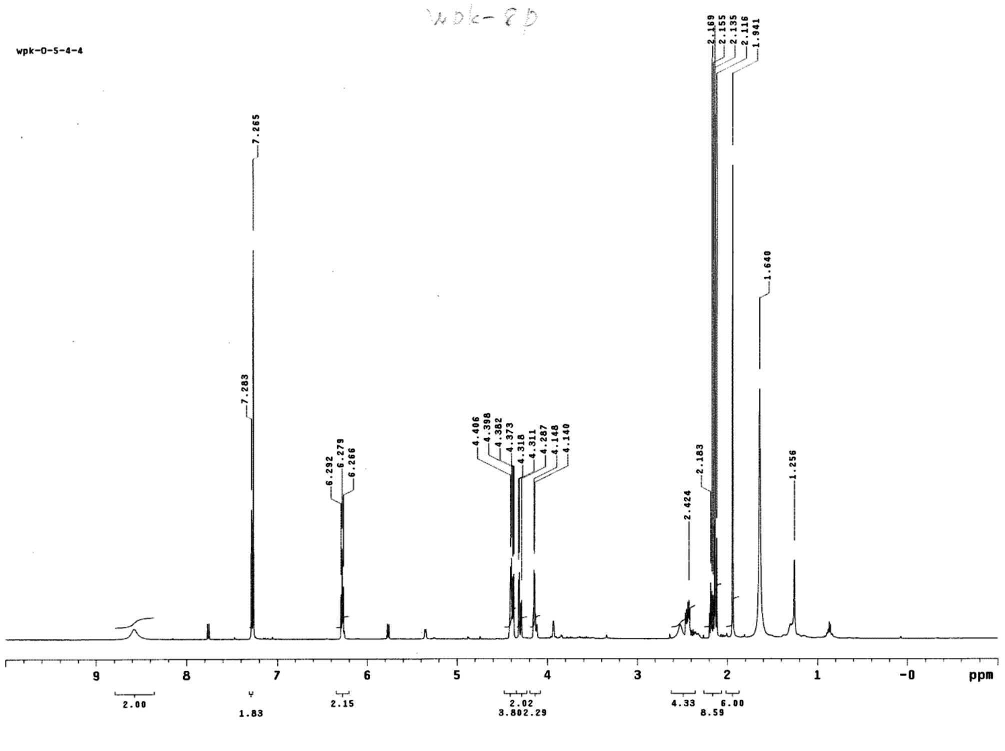

Figure S21. ^{1}H NMR (500 MHz, $CDCl_3$) spectrum of **6**

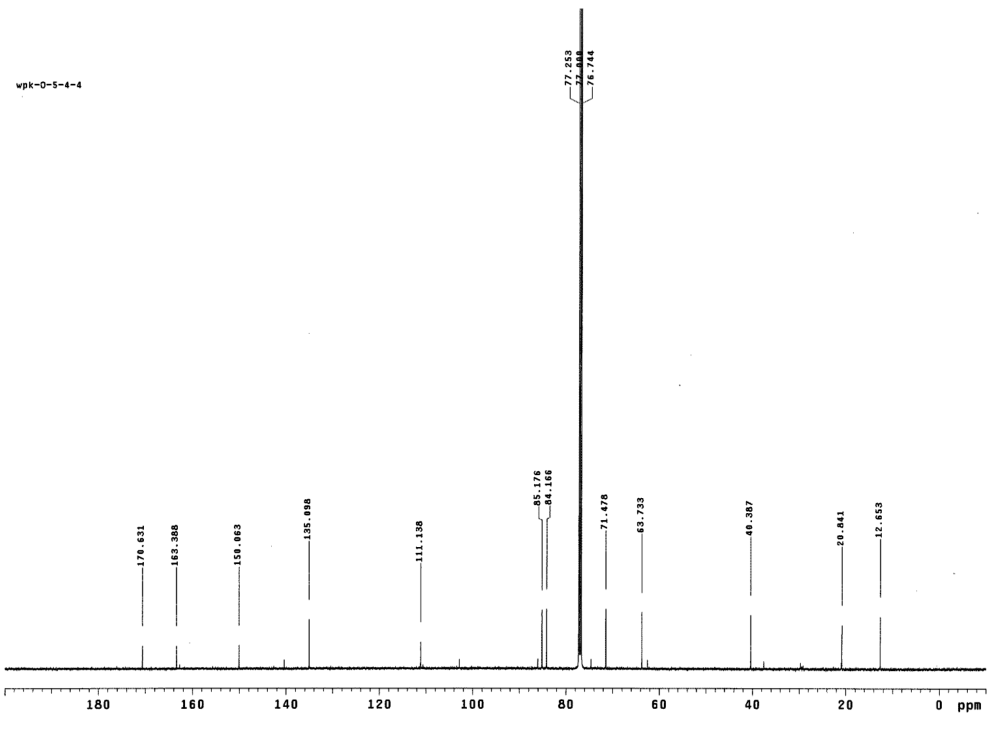

Figure S22. ^{13}C NMR (125 MHz, $CDCl_3$) spectrum of **6**

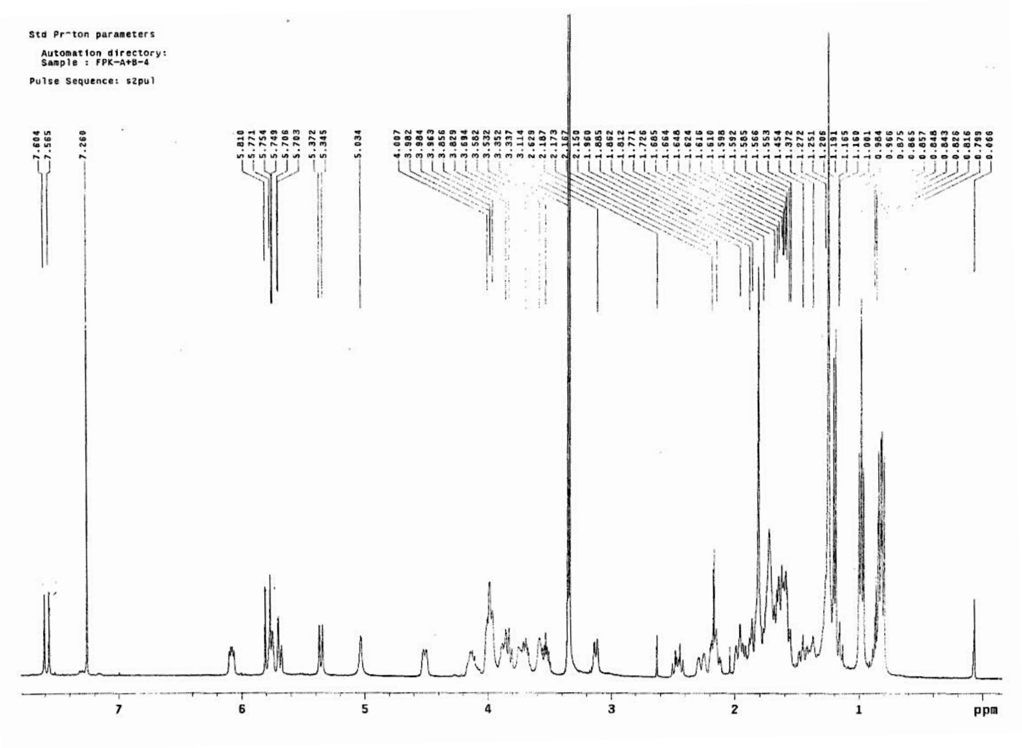

Figure S23. ^1H NMR (500 MHz, $CDCl_3$) spectrum of **7**

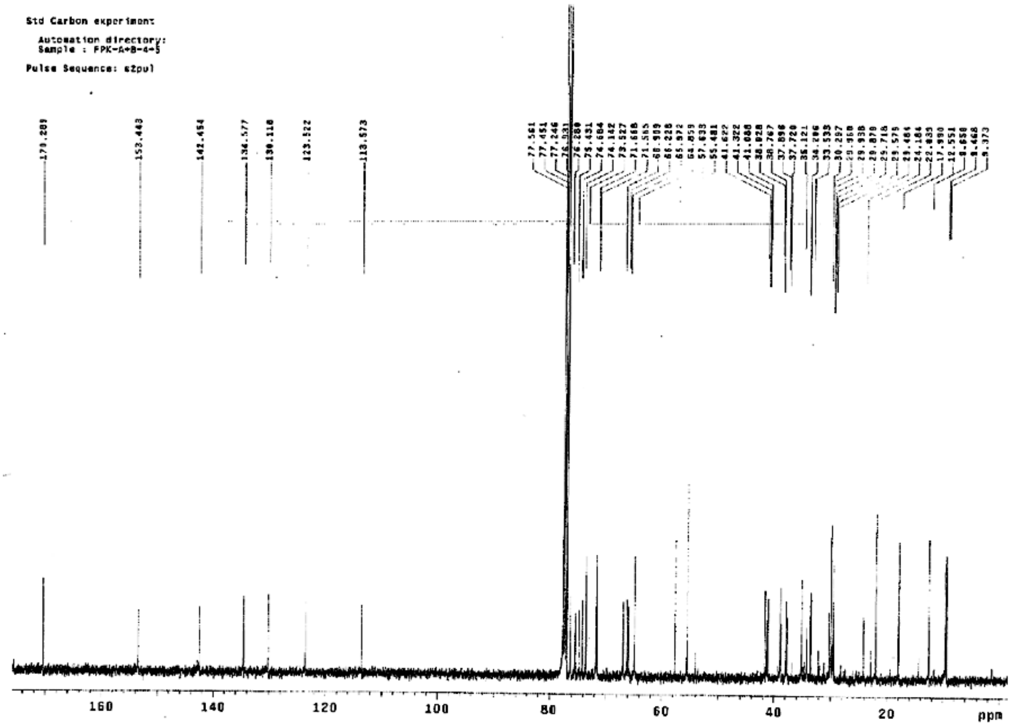

Figure S24. ^{13}C NMR (125 MHz, $CDCl_3$) spectrum of **7**
